# Gut microbiota and intestinal permeability in rheumatoid arthritis: therapeutic implications and future directions

**DOI:** 10.3389/fimmu.2026.1936364

**Published:** 2026-10-14

**Authors:** Jan Bilski, Agata I. Schramm-Luc, Marian Szczepanik, Piotr Pierzchalski, Kevin Luc

**Affiliations:** 1 Department of Biomechanics and Kinesiology, Chair of Biomedical Sciences, Institute of Physiotherapy, Faculty of Health Sciences, Jagiellonian University Medical College, Krakow, Poland; 2 Department of Internal and Agricultural Medicine, Faculty of Medicine, Jagiellonian University Medical College, Krakow, Poland; 3 Chair of Biomedical Sciences, Institute of Physiotherapy, Faculty of Health Sciences, Jagiellonian University Medical College, Krakow, Poland; 4 Department of Medical Physiology, Chair of Biomedical Sciences, Institute of Physiotherapy, Faculty of Health Sciences, Jagiellonian University Medical College, Krakow, Poland

**Keywords:** gut–joint axis, intestinal microbiota, intestinal permeability, microbial metabolites, mucosal immunity, physical activity, rheumatoid arthritis, short-chain fatty acids

## Abstract

Interactions between the intestinal microbiota, epithelial barrier and mucosal immune system have prompted investigation of gut-directed interventions in rheumatoid arthritis (RA). This review examines whether modifying these interactions can improve clinical outcomes and assesses the strength of the available evidence in RA. In experimental arthritis, interventions that preserve epithelial integrity or alter microbial metabolism can reduce disease severity, providing a biological basis for further study. However, their relevance to human RA remains uncertain, since effects observed in animal models do not establish clinical efficacy, and changes in intestinal measurements alone do not demonstrate improvement in joint disease. Future controlled trials should assess disease activity, and physical function alongside microbial composition and function, intestinal measurements and immune responses. Repeated sampling may help establish whether intestinal changes precede or accompany clinical improvement, while recording medication exposure and dietary intake is needed to interpret these relationships. Candidate biomarkers will require prospective validation before they can be used to select patients for treatment. These studies should determine whether gut-directed interventions provide additional clinical benefit, in whom, and at what risk.

## 1 Introduction

Rheumatoid arthritis (RA) is a chronic systemic autoimmune disease characterized by persistent synovial inflammation, which may lead to progressive joint destruction, disability and a considerable burden of extra-articular comorbidity (1). Although biologics and targeted synthetic disease-modifying antirheumatic drugs (DMARDs) have improved RA management, some patients do not achieve sustained remission, respond incompletely to treatment or develop intolerance to therapy (1–5). These observations have increased interest in biological processes outside the synovium that may contribute to the persistence of inflammation, influence therapeutic response and affect the long-term stability of remission (2).

The intestinal microbiota produces metabolites and other microbial products that interact with epithelial and immune cells. Alterations in microbial activity or epithelial integrity may modify mucosal immune responses and increase exposure to luminal antigens, providing a possible route by which intestinal events influence systemic inflammation and immune activation in the synovium (6–11). Evidence for this sequence comes mainly from experimental arthritis models, in which preservation of intestinal barrier function reduces disease severity. In mice, larazotide treatment was also associated with reduced trafficking of gut-primed T cells to the synovium (12); however, its relevance to human RA remains uncertain. Microbiome profiles differ according to disease stage, diet, geographical background, and analytical approach, while longitudinal studies indicate that at least some alterations emerge close to disease onset rather than remaining stable throughout the preclinical period (6, 13–17). These findings do not establish a single microbial disturbance as a cause of RA or a therapeutic target shared by all patients.Treatment exposure adds another source of uncertainty as DMARDs and commonly used concomitant medications may affect the microbial composition, intestinal function, or both (18–21). Changes detected during treatment may consequently result from direct drug effects, reduced inflammatory activity, or a combination of the two. Changes in proteins used as indirect markers of barrier function have been reported during anti-TNF treatment; however, these observations do not show that epithelial repair mediates the clinical response (22). The first part of this series, *Gut microbiota and intestinal permeability in rheumatoid arthritis*: *pathogenic mechanisms*, described the biological basis of gut–joint interactions and discussed their possible translational implications (11). The present review turns to therapeutic evidence and discusses whether interventions targeting these interactions have produced measurable benefits in patients with RA. Mechanistic findings are included where they clarify how an intervention may act, assist in the interpretation of a measurement or explain why an experimental finding may not translate into clinical practice.

The interventions discussed include dietary and prebiotic approaches, microbial metabolites, postbiotic preparations, probiotics and defined commensals, fecal microbiota transplantation, pharmacological protection of the intestinal barrier, and physical exercise. Their proposed sites of action within the intestine are shown in **Figure 1**. Evidence for each approach requires consideration of several distinct outcomes, including changes in the microbiome, metabolites, barrier function, and immune responses, as well as effects on disease activity, pain, and physical function. A change in microbial composition or a circulating marker does not necessarily result in clinical improvement, and a clinical response cannot be taken as evidence that the intervention acted through the gut. Therefore, these approaches are considered in relation to established DMARD-based care, with an emphasis on human intervention studies, safety, and the validation required before intestinal biomarkers can be used to guide treatment selection.

**Figure 1:**
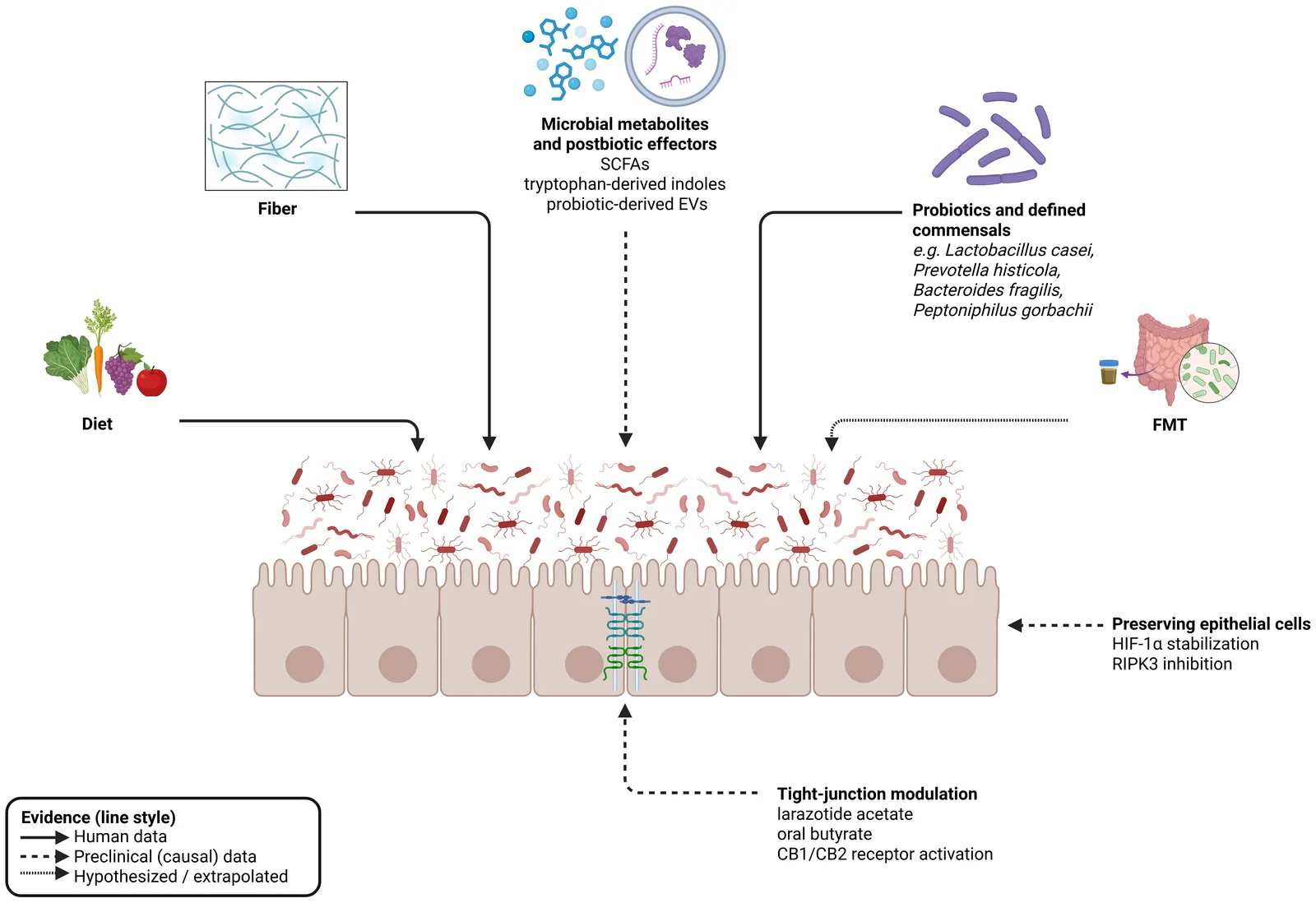
Potential therapeutic points of action in the gut–joint axis in rheumatoid arthritis. The figure summarizes gut-directed strategies discussed in this review, including dietary and fiber-based approaches, microbial metabolites and postbiotic effectors, probiotics and defined commensals, fecal microbiota transplantation, tight-junction modulation, and preservation of epithelial cell viability. Solid arrows indicate supporting human data; dashed arrows indicate preclinical evidence of causal effects in experimental systems; dotted arrows indicate hypothesized or extrapolated mechanisms. These designations describe the evidence supporting the depicted relationships and do not imply established clinical efficacy or demonstrated intestinal mediation of benefit in rheumatoid arthritis. CB1/CB2, cannabinoid receptors 1 and 2; EVs, extracellular vesicles; FMT, fecal microbiota transplantation; HIF-1α, hypoxia-inducible factor 1-alpha; RIPK3, receptor-interacting serine/threonine-protein kinase 3; SCFAs, short-chain fatty acids. Created with BioRender.com.

This narrative review was based on searches of PubMed/MEDLINE and Scopus, with the final search performed on 24 June 2026. Human studies were prioritized, while preclinical studies were included mainly to support the interpretation of proposed biological mechanisms.

## 2 Pharmacological reinforcement of intestinal barrier integrity

The observation that alterations in epithelial integrity may occur before the clinical appearance of experimental arthritis, together with evidence that improvement of barrier function can lessen disease severity in some models, has led to the suggestion that the intestinal barrier may be considered as a possible therapeutic target in RA (12, 23). At present, however, interventions directed at the intestinal barrier in RA remain largely experimental.

### 2.1 Targeting the paracellular pathway

Tight junctions (TJs) regulate the paracellular movement of water, ions, and small solutes between neighboring epithelial cells. Disturbance of this system may increase epithelial permeability and allow the mucosal immune system to encounter greater amounts of luminal antigens or microbial products (24). Larazotide acetate is a synthetic peptide acting locally at the intestinal epithelium, and has been studied mainly in celiac disease as a modulator of paracellular permeability (25).

In collagen-induced arthritis (CIA), Tajik et al. (12) showed that increased intestinal permeability and activation of zonulin-family signaling were already present before the onset of clinical arthritis. They also found that interventions introduced before disease onset reduced subsequent disease expression. In this model, oral butyrate, activation of cannabinoid receptors, and larazotide acetate improved epithelial barrier function and reduced the severity of arthritis (12). Larazotide treatment was also associated with decreased trafficking of gut-primed CD4^+^ T cells to the synovium, which suggests that events at the intestinal barrier may influence the movement of immune cells between the gut and the joint (12).

These observations indicate that reinforcement of the paracellular barrier can modify arthritis under experimental conditions (12). However, several points should be considered. Larazotide has not been tested in clinical trials in RA patients, and its relevance to human RA therefore remains unknown (12). It is also uncertain whether effects observed during the pre-arthritic phase of experimental disease can be transferred to established RA, in which systemic inflammation, previous and current drug exposure, and long-standing tissue remodeling may affect the intestinal barrier in other ways (12, 22, 26, 27). Measurements reported as circulating “zonulin” also require caution, since widely used commercial assays may detect proteins other than pre-haptoglobin 2, the protein originally identified as zonulin. These measurements should therefore not be interpreted as direct or specific measures of intestinal permeability (28, 29). Finally, the paracellular pathway should be distinguished from transcellular transport. Stabilization of TJs may reduce the passage of small molecules or soluble antigens between epithelial cells, but it should not be assumed to prevent active uptake, endocytosis, M-cell sampling, or transcytosis of larger antigens, microbial vesicles, or intact microorganisms (24). Larazotide and related TJ-directed approaches should therefore be regarded primarily as interventions aimed at paracellular permeability, rather than as general inhibitors of microbial-product exposure or other routes of epithelial antigen transport (24). Larazotide acetate remains the most extensively studied agent in this class; however, its pivotal Phase 3 trial in celiac disease failed to meet its primary endpoint and was discontinued, and no human RA trial data exist (30).

### 2.2 Preservation of epithelial cell survival: the HIF-1α–RIPK3 axis

Impairment of intestinal barrier function may result not only from altered regulation of TJs but also from epithelial injury and loss of epithelial cells from the mucosal surface. This is clinically relevant, since therapeutic approaches aimed mainly at strengthening TJs may be insufficient when barrier disruption is accompanied by active epithelial cell death (31). Recent experimental evidence has indicated that necroptosis of intestinal epithelial cells may contribute to the loss of barrier integrity observed in inflammatory arthritis (32). Under physiological oxygen conditions, hypoxia-inducible factor 1-alpha (HIF-1α) is hydroxylated by prolyl hydroxylase domain enzymes, which leads to its targeting for proteasomal degradation. Inhibition of these enzymes stabilizes HIF-1α and may influence the epithelial response to stress (33). In a CIA model, pharmacological stabilization of HIF-1α by the prolyl hydroxylase inhibitor roxadustat preserved epithelial barrier integrity and reduced both the incidence and severity of arthritis in mice (32). In the same experimental model, inhibition of receptor-interacting serine/threonine-protein kinase 3 (RIPK3) protected the intestinal epithelium and reduced the manifestations of arthritis (32). Expression of RIPK3 was also found to be enhanced in the intestinal epithelium of patients with new-onset RA, providing an initial human correlate for these preclinical observations (32). These data suggest that epithelial necroptosis may represent a potential therapeutic target in experimental arthritis (32). However, several points should be considered before this mechanism is viewed in clinical terms.

Roxadustat is used to treat symptomatic anemia associated with chronic kidney disease, where its systemic action stimulates erythropoiesis. In RA patients without anemia, stimulation of erythropoiesis would be an unwanted systemic effect of treatment intended to protect the intestinal barrier. Hypertension and thrombotic events reported during clinical use raise further safety concerns (34, 35), particularly in patients with RA, who already have an increased cardiovascular risk (36). The beneficial effects observed in experimental arthritis therefore provide no justification for systemic roxadustat treatment solely to preserve intestinal barrier function (34, 35). For this mechanism to be pursued clinically, the epithelial effects would need to be separated from systemic HIF activation. The safety and efficacy of such an approach would then require evaluation in patients with RA. Inhibition of RIPK3 also remains experimental and cannot currently be considered a clinically available barrier-directed therapy (37).

### 2.3 Effects of standard RA treatment on the intestinal environment

Standard RA treatment can influence the intestinal environment as well as the measurements used to characterize it. Nonsteroidal anti-inflammatory drugs can increase intestinal permeability and cause small-intestinal inflammation; intestinal abnormalities observed in patients with RA may therefore partly reflect NSAID exposure rather than the disease itself (38–40). Methotrexate acts directly on bacterial growth and metabolism, and longitudinal studies in patients with RA, supported by experimental work, have linked its use to changes in the microbiota and host immune activation. However, these observations do not show that intestinal permeability is restored during treatment (41). Glucocorticoid exposure also needs to be considered, although its microbial effects cannot be assumed to be uniform. In one longitudinal RA cohort, prednisone use was not independently associated with overall gut microbial community composition after adjustment for measured covariates (42). Effects related to dose, duration or particular microbial functions remain possible. For targeted therapies, much of the distinction rests on what was measured and in which setting. During anti-TNF treatment, serum occludin and claudin-1 concentrations moved towards control values in patients achieving remission, but these indirect measurements did not establish that changes in the intestinal barrier accounted for the clinical response (22). In a small additional group of five patients treated with the IL-6 receptor inhibitor tocilizumab, serum LBP and sCD14 decreased after three months alongside clinical response (43). These markers reflect both exposure to microbial products and the host inflammatory response. Their reduction may therefore reflect the anti-inflammatory effects of IL-6 receptor blockade and does not, by itself, demonstrate reduced microbial translocation or restored intestinal permeability. Tofacitinib, a JAK inhibitor, altered gut microbial composition and mucosal immune populations in experimental arthritis, providing evidence of intestinal effects in that setting but not of barrier restoration in patients with RA (44). Findings obtained with one targeted therapy should not be assumed to apply to other agents, particularly when the evidence comes from different experimental models or patient populations. Intestinal findings obtained during treatment therefore need to be interpreted in relation to disease activity, the medication used, its dose and duration, and any recent treatment changes. Antibiotic and proton-pump inhibitor exposure should be documented as well. Distinguishing direct effects of a drug from changes that accompany control of inflammation requires longitudinal assessment. Neither a change in microbial composition nor a reduction in circulating inflammatory or translocation-related markers is sufficient on its own to demonstrate that the therapeutic response is mediated through the gut.

### 2.4 Clinical perspective and limitations

Future clinical studies of barrier-directed treatments may be more informative if they enrol patients with evidence of intestinal barrier dysfunction rather than unselected RA populations (6, 16). Such characterization should not treat barrier dysfunction as a single abnormality. Increased paracellular permeability, epithelial injury and transcellular antigen transport may occur together, but they represent distinct processes and may not respond to the same intervention (24, 31). It remains unknown whether any of these abnormalities can identify patients who are more likely to benefit from treatment.

## 3 Diet, microbial metabolites, and postbiotic effectors

### 3.1 Diet

Diet is one of the most accessible environmental factors that can modify the intestinal ecosystem (45–47). It provides substrates for microbial metabolism, affects the production of short-chain fatty acids (SCFAs) and other bioactive molecules, and may influence epithelial barrier function, mucosal immune activity, and systemic inflammation (45, 47–49). In RA, diet is therefore relevant not as an alternative to established disease-modifying treatment, but as a factor that may modify biological processes involved in persistent inflammation or treatment response in selected patients (49–51).

Nutritional interventions in RA should be viewed as adjunctive approaches that may act through several related mechanisms. These include improvement of general metabolic health, reduction of cardiovascular risk, changes in body composition, modification of dietary fat quality, increased intake of fermentable substrates, and altered production of microbial metabolites (45, 50). A contribution of the gut microbiota to these effects is biologically plausible, although it remains difficult to distinguish microbial mechanisms from other consequences of dietary change (47, 51).

Within the gut–joint axis, the dietary components of greatest interest are those that affect microbial function rather than microbial composition alone (48, 51). Fermentable fibers can be converted by intestinal bacteria into SCFAs, particularly acetate, propionate, and butyrate (46, 48, 51, 52). Polyphenols and other plant-derived compounds may also be metabolized by the microbiota into smaller bioactive molecules (45, 53, 54). Dietary tryptophan can serve as a precursor for microbial indole derivatives, some of which interact with aryl hydrocarbon receptor (AhR)-dependent pathways (51, 55). These examples indicate that diet should not be interpreted only in terms of nutrient intake, but also as a means of modifying microbial metabolic output (51).

However, this field is still limited by important methodological problems. Dietary studies in RA differ in disease duration, baseline treatment, background diet, adherence, disease activity, body weight, and outcome measures (56). In observational studies, dietary patterns may also reflect differences in physical activity, socioeconomic circumstances and other health behaviors, so their associations with RA outcomes cannot be attributed to diet alone. Many studies have also assessed clinical symptoms or inflammatory markers without parallel analysis of microbiome function, intestinal permeability, or metabolite profiles (45, 47, 50, 57). The available data therefore support diet as a plausible adjunctive approach, but not as a uniformly effective gut-targeted therapy for all patients with RA (45).

Future dietary studies in RA should therefore move beyond general nutritional advice and use better-defined interventions linked to outcomes related to the microbiota, metabolism, and the intestinal barrier (50).

Several dietary patterns have been studied in RA, although the quality and depth of evidence differ substantially (56, 58). Mediterranean-style diets, vegetarian or vegan diets, and fiber-rich approaches are particularly relevant to the gut–joint axis, because they increase the intake of plant-derived substrates and may support microbial metabolic activity (56, 58). These patterns should not, however, be treated as equivalent interventions, since they differ in composition, feasibility, nutritional consequences, and probable mechanisms of action (45, 50, 51, 56, 59, 60).

The Mediterranean diet is characterized by a high intake of vegetables, fruits, legumes, whole grains, nuts and olive oil, moderate intake of fish, and limited intake of red meat and highly processed foods (56, 57, 61–63). This pattern is relevant to RA because it combines several potentially beneficial dietary elements, including fiber, monounsaturated fatty acids, omega-3 fatty acids, and polyphenols (57, 61–63). In clinical studies, Mediterranean-style dietary interventions have been associated with improvements in some measures of RA disease activity and inflammatory status, and a cross-sectional study associated greater Mediterranean diet adherence with lower disease activity and differences in gut microbiota composition (57, 61, 62, 64). However, the Mediterranean diet should be interpreted as a complex dietary pattern rather than a microbiome-specific intervention as its effects may reflect combined changes in fat quality, antioxidant intake, body weight, cardiometabolic risk, and microbial metabolism (57, 61, 62).

Vegetarian and vegan diets have also been studied in RA, including older intervention studies which suggested improvement in selected clinical outcomes in some patients (65). More recent evidence comes from the “Plants for Joints” randomized controlled trial, in which a 16-week multidisciplinary lifestyle program based on a whole-food plant-based diet, physical activity, and stress management substantially reduced disease activity and improved metabolic status in RA patients with low-to-moderate disease activity (66). This study supports the view that plant-based dietary strategies may have clinical value when implemented as part of a structured lifestyle intervention, although the effect cannot be attributed to diet alone because exercise and stress management were integral components of the program. In this trial, background antirheumatic medication was intended to remain stable for three months before and during the trial whenever possible, although medication exposure varied between participants (66). These diets may increase fiber intake and reduce intake of animal fat, while also changing the exposure to dietary antigens, salt, saturated fatty acids, and processed foods (67, 68). Some protocols have included fasting or elimination phases, which complicates interpretation of the specific contribution of the vegan diet itself (65, 69). In addition, strict vegan diets require nutritional planning because deficiencies in vitamin B12, iron, calcium, iodine, vitamin D, or omega-3 fatty acids may occur if supplementation and dietary quality are inadequate (70). Therefore, although vegan or vegetarian approaches may be useful for selected motivated patients, they cannot be recommended as a universal RA strategy without individual nutritional assessment (65, 71).

Fiber-rich diets represent a more functionally defined approach because fermentable fiber provides substrate for bacterial production of SCFAs (6, 72, 73). This makes fiber particularly relevant to the therapeutic rationale of the gut–joint axis (68, 72, 74). In RA, high-fiber dietary supplementation has been associated in a feasibility study with changes in regulatory immune parameters and reductions in selected intestinal or inflammatory biomarkers (72). These findings support the concept that increasing fermentable substrate availability may influence immune regulation, but they do not demonstrate that fiber supplementation alone is sufficient to control RA disease activity (72, 75).

A comparison of these dietary patterns suggests that their therapeutic value lies in different but overlapping domains (59, 76). Mediterranean diets may be most suitable as a long-term, cardio metabolically favorable dietary pattern for patients with RA (57, 61, 63, 64). Vegan or vegetarian diets may benefit selected individuals, but their interpretation is complicated by restriction, adherence, and nutritional adequacy (70, 74). Fiber-rich diets and prebiotic strategies provide the most direct link to microbial metabolite production, but their clinical use requires attention to dose, tolerability, and baseline microbiome function (72, 73). Thus, the most defensible conclusion is not that one diet should be prescribed to all patients with RA, but that dietary interventions should be selected according to feasibility, nutritional safety, comorbidity profile and, in future studies, microbial and metabolic biomarkers.

### 3.2 Prebiotic and fiber supplementation

Prebiotic and fiber-based interventions represent a more defined approach than broad dietary patterns because they aim to increase the availability of substrates for microbial fermentation (8, 77). In this context, dietary fiber should not be viewed only as an indigestible component of food, but also as a source of microbiota-accessible carbohydrates which can be metabolized by intestinal bacteria into SCFAs and other bioactive products (77–79). This provides a direct conceptual link between diet, microbial function and host physiology, and makes fiber supplementation particularly relevant to the therapeutic discussion of the gut–joint axis in RA (8, 77).

The term prebiotic should be used with some precision. Not every fiber source fulfils the criteria for a prebiotic, and not all prebiotics exert the same biological effects. Inulin, fructo-oligosaccharides, galacto-oligosaccharides, resistant starch and selected plant polysaccharides differ in fermentability, site of metabolism, tolerability and effects on microbial ecology (78, 80, 81). Therefore, fiber supplementation in RA should not be discussed as a single intervention. It is more accurate to regard it as a family of substrate-based strategies which may increase microbial metabolite production in individuals whose baseline microbiota retains the capacity to use these substrates effectively (81).

Early clinical data support the feasibility of this approach, but do not yet establish fiber supplementation as a disease-modifying intervention in RA. In a 28-day pilot study of patients with RA, high-fiber supplementation was associated with an increase in circulating regulatory T (Treg) cells (CD4^+^CD25^+^FoxP3^+^ cells) and an improved Th1/Th17 ratio, together with reductions in selected serum biomarkers, including zonulin and calprotectin (72). The immune findings suggest a change in T-cell regulation, but the biomarker changes do not establish restoration of intestinal barrier function. In particular, zonulin measurements require caution because of the limited specificity of commonly used assays (28, 29, 72). However, the short duration, modest sample size and feasibility design of the study limit the strength of the conclusions (72).

A more recent clinical signal comes from the Superfibre study, presented in abstract form, in which patients with active RA were randomized to receive inulin or placebo in addition to background conventional synthetic DMARD therapy (82). According to the available abstract, fiber supplementation was associated with a higher likelihood of clinical response and changes in peripheral Th17/Treg balance, with the most apparent benefit reported in patients receiving methotrexate (82). These observations are important because they suggest that fiber supplementation may interact with established pharmacotherapy rather than act as an independent treatment. Nevertheless, because the data are currently available only as a conference abstract, they should be considered hypothesis-generating until the full trial methodology, microbiome analyses, adverse events and complete clinical outcomes are published (82).

This possible interaction between prebiotic supplementation and methotrexate is of particular translational interest (21). Methotrexate remains the anchor conventional synthetic DMARD in RA, but response varies between patients (21, 83). If it were proven that microbial metabolites derived from dietary fiber influence the regulation of mucosal immunity or drug-induced changes in the microbiome, prebiotic supplementation could in the future become the subject of research as a strategy aimed at improving treatment response in specific patient groups; however, at present, this should be treated as a hypothesis to be verified (21, 82).The clinical use of fiber also requires practical caution. Rapid escalation of fermentable fiber may cause bloating, abdominal discomfort and reduced adherence, particularly in patients with coexisting functional gastrointestinal symptoms (77, 84). Moreover, the same prebiotic substrate may have different metabolic consequences depending on the resident microbiota, background diet and medication exposure (85). Therefore, future trials should report not only disease activity outcomes, but also tolerability, adherence, dietary background, microbiome composition, metabolite production and barrier-related biomarkers (82, 83).

In a CIA model, an RA-derived strain of *P. copri* promoted fermentation of dietary fiber to succinate and fumarate, and a fiber-containing diet aggravated intestinal inflammation and arthritis (86). These findings suggest that the response to fiber may depend on the metabolic activity of the resident microbiota, but do not establish how microbiome measurements should guide supplementation in patients with RA.

Current evidence suggests that supplementation with prebiotics and fiber may be a possible adjunctive strategy in the treatment of RA, but not as a standalone therapy. The rationale is to increase the availability of substrates for microbial metabolite production, but whether these changes lead to sustained clinical benefit in RA remains uncertain (16, 87, 88). Future studies should determine whether baseline microbial composition, fecal SCFA concentrations, dietary intake, intestinal permeability markers or methotrexate exposure identify patients more likely to respond to fiber-based interventions (82).

Dietary and targeted nutritional interventions are compared in **Table 1**, with particular attention to their mechanistic rationale, clinical relevance and current weight of evidence.

**Table 1:** Dietary patterns and targeted nutritional interventions in rheumatoid arthritis.

Intervention	Key finding/claim	Weight of evidence	Studies (supporting or refuting)	References
Mediterranean Diet	Greater adherence to a Mediterranean diet has been associated with lower RA incidence and, in patients with RA, lower disease activity and differences in gut microbial composition. These observations do not establish that microbiota changes mediate clinical benefit.	Moderate	Hu et al. (2025) 9-year cohort (117,341 UK Biobank participants) + systematic review: higher Mediterranean diet adherence was significantly associated with reduced RA incidence. Inverse association confirmed across multiple observational study designs.	(57)
			Picchianti Diamanti et al. (2020) Cross-sectional study of 60 consecutive RA patients receiving stable RA treatment for at least 3 months. Patients were stratified by PREDIMED score. High MD adherence (n=20) associated with significantly lower CRP (p=0.037) and DAS28 (p=0.034); 16S microbiota analysis revealed distinct compositional profiles correlated with adherence level, linking dietary pattern directly to gut microbial ecology in RA.	(64)
Whole-Food Plant-Based Lifestyle Intervention	A combined program of plant-based diet, physical activity and stress management reduced RA disease activity and improved metabolic status. The contribution of diet alone and any mediation through the gut microbiota remain uncertain	Moderate	Walrabenstein et al. (2023) Assessor-blind, 16-week RCT; 83 participants randomized and 77 analyzed. The intervention combined a whole-food plant-based diet, physical activity and stress management. Background antirheumatic medication was intended to remain stable for three months before and during the trial whenever possible; treatment exposure was heterogeneous. DAS28 improvement was 0.9 points greater than in the control group (95% CI 0.4–1.3; p<0.0001). Additional significant improvements were observed in body weight, fat mass, LDL cholesterol and HbA1c. The contribution of diet alone could not be isolated, and mediation through the gut microbiota or intestinal barrier was not established.	(66)
			Wagenaar et al. (2024) One-year observational extension of the *Plants for Joints* (66) RCT; 65 of 77 RA participants completed follow-up. The original control group also received the multidisciplinary lifestyle intervention before entering follow-up; therefore, the extension did not retain a randomized untreated control group. Antirheumatic medication tapering was permitted in participants with DAS28 <2.6. Improvements in DAS28 and selected cardiometabolic outcomes were sustained one year after intervention completion. These findings suggest sustained benefits of the combined lifestyle program, but the contribution of diet alone could not be isolated, and mediation through the gut microbiota or intestinal barrier was not established.	(290)
Fiber and prebiotic supplementation	Fermentable fiber may support SCFA production and immune regulation, but clinical evidence is still limited. Whether baseline microbial or metabolic profiles can identify patients more likely to benefit from fiber supplementation remains to be established.	Moderate	Hager et al. (2019) A 28-day pilot feasibility study enrolled 36 patients with RA who continued stable DMARD therapy throughout the intervention. High-fiber supplementation significantly increased CD4+CD25+FoxP3+ Tregs and improved Th1/Th17 ratio; reduced serum ZFP and serum calprotectin. No disease activity primary endpoint; no conclusions on clinical efficacy can be drawn.	(72)
			Durholz et al. (2020) Short-term, uncontrolled fiber supplementation study in patients with RA. Patients were in clinical remission and continued their established, stable conventional synthetic, targeted synthetic or biological DMARD therapy throughout the study. Serum SCFA concentrations increased following supplementation. The absence of a randomized control group limits causal interpretation, and these metabolic changes do not establish clinical efficacy in RA.	(73)
	Findings reported in a conference abstract provide preliminary evidence. The limited methodological detail prevents a full assessment of study quality, and the reported clinical effects require confirmation in a full publication.	Emerging	Daien et al. (2025) Superfiber trial (EULAR 2025 abstract) - Inulin vs placebo on background csDMARD therapy: inulin group showed higher ACR20/50 response rate and improved Th17/Treg ratio; effect most pronounced in methotrexate-receiving subgroup. Full peer-reviewed publication pending.	(82)
	A high-fiber diet worsened experimental arthritis in mice colonized with an RA-derived *P. copri* strain. This finding suggests that microbial context may influence the response to fiber, but does not establish a basis for selecting or excluding patients from supplementation.	Emerging	Jiang et al. (2022) CIA model with RA patient-derived *P. copri* RA strain: colonization enhanced complex carbohydrate catabolism through expanded carbohydrate utilization loci; overproduction of succinate and fumarate activated proinflammatory macrophages via succinate dehydrogenase signaling; high-fiber diet worsened CIA severity and intestinal inflammation vs *P. copri*-free controls; succinate supplementation alone was sufficient to exacerbate CIA, confirming it as the primary effector metabolite driving the observed exacerbation (86).	(86)

Weight of evidence is a descriptive assessment of the evidence supporting the stated finding or claim, taking into account study design, methodological limitations, consistency and relevance to RA. Strong denotes consistent evidence from adequately conducted human studies; Moderate denotes human evidence limited by study size, design or inconsistent findings; Emerging denotes preliminary evidence from experimental models, case reports or conference abstracts. Indirect identifies evidence from human populations without RA that is extrapolated to RA. These categories do not constitute a formal GRADE assessment. The rating applies to the outcome described: evidence of clinical benefit does not establish mediation through the gut microbiota or intestinal barrier, and changes in intestinal or circulating biomarkers do not by themselves demonstrate clinical efficacy.

ACR20/50, American College of Rheumatology 20%/50% response criteria; CD4^+^CD25^+^FoxP3^+^ Tregs, regulatory T cells expressing CD4, CD25 and FoxP3; CIA, collagen-induced arthritis; CRP, C-reactive protein; csDMARDs, conventional synthetic disease-modifying antirheumatic drugs; DAS28, Disease Activity Score in 28 joints; EULAR, European Alliance of Associations for Rheumatology; FoxP3, forkhead box P3, a transcription factor associated with regulatory T cells; HbA1c, glycated hemoglobin; LDL, low-density lipoprotein; MD, Mediterranean diet; *P. copri*, *Prevotella copri*; PREDIMED score, Mediterranean diet adherence score derived from the PREDIMED dietary assessment tool; RA, rheumatoid arthritis; RCT, randomized controlled trial; SCFAs, short-chain fatty acids; Th1/Th17, T helper 1/T helper 17 cells; Th17/Treg ratio, ratio of T helper 17 cells to regulatory T cells; UK Biobank, United Kingdom Biobank; ZFP, zonulin-family proteins/peptides.

### 3.3 Short-chain fatty acids as microbial effector molecules

Short-chain fatty acids represent one of the most direct links between diet, microbial metabolism and host physiology (52). They are generated mainly through bacterial fermentation of non-digestible carbohydrates, including dietary fiber and resistant starch, and include acetate, propionate, and butyrate (52). Although these metabolites are often discussed together, they differ in their sites of production, tissue distribution, and biological activity (52, 89, 90). In the context of RA, their relevance lies in their potential to modify epithelial barrier function, mucosal immune regulation, and processes involved in joint inflammation and bone erosion (91, 92).

**Table 2** summarizes key preclinical and translational evidence for microbial metabolite– and postbiotic-based effectors in RA, including SCFAs/butyrate, tryptophan-derived indoles acting through AhR, and probiotic-derived extracellular vesicles, together with the weight of evidence and supporting studies for each.

**Table 2:** Microbial metabolites and postbiotic effectors.

Intervention	Key finding/claim	Weight of evidence	Studies (supporting or refuting)	References
SCFAs/butyrate	In experimental models, butyrate influences regulatory T cell differentiation and macrophage polarization. SerBut (O-butyryl-L-serine) improved oral bioavailability and reduced arthritis severity in mice; its pharmacokinetics and clinical efficacy in humans remain to be established.	Emerging	Cao et al. (2024) CIA and EAE mouse models: SerBut showed significantly higher oral bioavailability vs plain sodium butyrate; reduced arthritis and neuroinflammation at lower doses; boosted Foxp3^+^ Tregs; shifted macrophage polarization toward M2 anti-inflammatory phenotype; modulated the gut–immune axis. No human pharmacokinetic or clinical data exist.	(101)
Tryptophan-derived indoles/AhR activation	CIA and epithelial-cell studies support AhR-dependent effects; human RA data are associative metabolomic findings.	Emerging	Moulin et al. (2024) Targeted Trp metabolomics in 574 untreated RA patients (ESPOIR) + 98 healthy controls: significantly decreased KYNA, XANA, and indole derivatives; elevated QUIN. Metabolite levels correlated with DAS28, IL-6, and quality of life. In a separate experimental component, AADAT enzyme supplementation was protective in CIA model. This finding supports further investigation but does not establish therapeutic efficacy in patients with RA.	(112)
			Wu et al. (2026) CIA model; Caco-2 intestinal epithelial cell *in vitro* model Gut microbiota-derived ILA and IAA reduced joint inflammation via AhR-mediated Treg expansion; microbiota depletion reduced indole metabolite availability and worsened CIA; AhR dependency validated with the antagonist CH223191.	(107)
			Su et al. (2024) CIA model IAA attenuated CIA model through AhR-TAZ-Tip60 pathway; TAZ acetylation prevented ubiquitin-mediated Foxp3 degradation, stabilizing Tregs and suppressing Th17 differentiation — identifying a specific post-translational epigenetic mechanism by which indole metabolites regulate Treg stability.	(108)
			Rosser et al. (2020) CIA model AhR activation in regulatory B cells via microbiota-derived tryptophan indoles amplified AhR-dependent IL-10 production, supporting a broader AhR–Breg–Treg immunological circuit that extends beyond the epithelial barrier effects described above.	(291)
Probiotic-derived extracellular vesicles	EVs may act as cell-free microbial effectors, but standardization and safety remain unresolved.	Emerging	Dell’Atti et al. (2025) Review: *Lactobacillus* spp.-derived EVs suppress NF-κB and MAPK signaling in macrophages and RA-FLS *in vitro*; EVs from L. reuteri suppress osteoclastogenesis in bone marrow cell models; EVs from L. plantarum restore epithelial barrier function in inflamed monolayers. No clinical RA trials of probiotic-derived EVs exist.	(124)

Weight of evidence is a descriptive assessment of the evidence supporting the stated finding or claim, taking into account study design, methodological limitations, consistency and relevance to RA. Strong denotes consistent evidence from adequately conducted human studies; Moderate denotes human evidence limited by study size, design or inconsistent findings; Emerging denotes preliminary evidence from experimental models, case reports or conference abstracts. Indirect identifies evidence from human populations without RA that is extrapolated to RA. These categories do not constitute a formal GRADE assessment. The rating applies to the outcome described: evidence of clinical benefit does not establish mediation through the gut microbiota or intestinal barrier, and changes in intestinal or circulating biomarkers do not by themselves demonstrate clinical efficacy. AADAT, aminoadipate aminotransferase; AhR, aryl hydrocarbon receptor; Breg, regulatory B cell; Caco-2 cells, human colorectal adenocarcinoma-derived epithelial cell line; CH223191, pharmacological antagonist of AhR; CIA, collagen-induced arthritis; DAS28, Disease Activity Score in 28 joints; EAE, experimental autoimmune encephalomyelitis; ESPOIR, French early arthritis cohort; EVs, extracellular vesicles; Foxp3/FoxP3, forkhead box P3; IAA, indole-3-acetic acid; ILA, indole-3-lactic acid; IL-6, interleukin-6; IL-10, interleukin-10; KYNA, kynurenic acid; MAPK, mitogen-activated protein kinase; M2 macrophages, alternatively activated macrophages; NF-kB, nuclear factor kappa-light-chain-enhancer of activated B cells; QUIN, quinolinic acid; RA, rheumatoid arthritis; RA-FLS, rheumatoid arthritis fibroblast-like synoviocytes; SCFAs, short-chain fatty acids; SerBut, O-butyryl-L-serine, a butyrate prodrug; TAZ, transcriptional coactivator with PDZ-binding motif; Th17, T helper 17 cells; Tip60, Tat-interactive protein 60 kDa, a histone acetyltransferase; Tregs, regulatory T cells; Trp, tryptophan; XANA, xanthurenic acid.

Butyrate has received the greatest attention because of its role in maintaining intestinal epithelial homeostasis. It is an important energy substrate for colonocytes and may support epithelial barrier integrity by influencing TJ proteins, epithelial metabolism, and inflammatory signaling (52, 93, 94). Experimental studies have shown that butyrate can affect host responses through several pathways, including histone deacetylase inhibition and activation of G protein-coupled receptors expressed on epithelial and immune cells (90, 94, 95). These observations provide a plausible explanation for why the loss of butyrate-producing bacteria or reduced butyrate availability may be biologically relevant in RA (92, 94, 96).

The immunological effects of butyrate are also relevant to the therapeutic interpretation of the gut–joint axis. Butyrate has been reported to support regulatory T cell differentiation and function, while limiting inflammatory T cell responses in experimental systems (92, 97). In RA, this is important because dietary or prebiotic strategies that increase butyrate production may contribute to a more regulatory mucosal environment. Its clinical significance in patients with RA remains to be established (96).

These mechanisms, reviewed in Part I (11), provide a plausible explanation for why the loss of butyrate-producing bacteria or reduced butyrate availability may be biologically relevant in RA (92, 94, 96).

Experimental studies suggest that the effects of butyrate extend beyond the intestinal epithelium. In models of inflammatory arthritis, butyrate has been reported to reduce osteoclastogenesis and bone erosion, with proposed mechanisms including inhibition of RANKL–TRAF6–NFATc1 signaling and reduced autoantibody production (98). Butyrate may also support intestinal barrier integrity through effects on tight-junction proteins, thereby reducing epithelial barrier dysfunction, microbial-product exposure and systemic inflammation (98). These experimental findings provide a rationale for investigating butyrate-related interventions, but do not establish clinical efficacy in patients with RA. Most evidence linking butyrate to osteoclastogenesis and joint protection remains experimental, and no butyrate-based therapy is available for RA (98).

Increasing the endogenous production of SCFAs through diet or prebiotic supplementation represents one way of modifying microbial metabolism (99). Another approach is to administer microbial metabolites, or metabolite-derived compounds, in a more pharmacologically defined form. This strategy is attractive because it may partly avoid the dependence on a patient’s resident microbiota to generate sufficient amounts of the required metabolite (98). However, direct administration also raises important problems related to formulation, absorption and tissue exposure. Butyrate has an unpleasant odor and taste, is rapidly absorbed and metabolized, and may not reliably reach the intended intestinal compartment when given in simple oral preparations (98, 100, 101).

These limitations are relevant in RA because the therapeutic aim would not simply be to increase luminal butyrate, but to deliver a biologically active signal to epithelial, immune or joint-related compartments involved in inflammatory persistence (98, 101). Direct sodium butyrate supplementation and fiber-derived butyrate production should therefore not be regarded as equivalent interventions (102). The former represents a pharmacological exposure influenced by formulation and distribution, whereas the latter depends on dietary substrate availability, microbial enzymatic capacity, epithelial absorption and host metabolism. For this reason, metabolite-delivery strategies require separate evaluation from dietary or prebiotic interventions (98, 101, 102).

Butyrate prodrugs have been developed to address some of these problems by chemically modifying butyrate to improve delivery, tolerability or systemic exposure. In principle, such compounds may provide a more reproducible way of increasing butyrate-related signaling than dietary modulation alone (101, 103). However, a prodrug does not reproduce the full biological setting of microbial fermentation, which includes local pH changes, cross-feeding interactions, co-produced metabolites and changes in microbial community structure (101, 104). Butyrate prodrugs should therefore be considered metabolite-based pharmacological tools, rather than substitutes for a functionally balanced intestinal microbiota.

SerBut is a serine-conjugated butyrate prodrug designed to improve the pharmacological delivery of butyrate. The rationale behind this compound is that conjugation of butyrate to an amino acid carrier may improve oral bioavailability and provide a more controlled exposure than direct butyrate administration (101). This makes SerBut relevant to the therapeutic discussion of the gut–joint axis, because it represents an attempt to translate a microbial metabolite signal into a defined pharmacological intervention (101). In experimental studies, SerBut has been reported to suppress autoimmune arthritis and neuroinflammation in mice (101). In the arthritis model, treatment was associated with reduced disease progression and immunological changes consistent with a more regulatory profile, including effects on Treg cells and macrophage polarization (101). These observations support the idea that butyrate-related pathways can be manipulated pharmacologically and not only through diet or microbiota modulation (101).

However, improvement of experimental arthritis in mice does not mean that the same compound would be effective in established, treatment-exposed and clinically heterogeneous human RA (101). At the same time, it illustrates why future metabolite-directed therapies should be studied with appropriate biomarker support, including measurement of butyrate exposure, intestinal barrier markers, immune readouts, and standard RA disease activity outcomes (98, 101).

### 3.4 Tryptophan-derived metabolites and AhR-dependent barrier regulation

Tryptophan-derived metabolites represent another group of microbial products with potential relevance to barrier regulation and mucosal immune control (105, 106). In contrast to SCFAs, which are generated mainly through bacterial fermentation of non-digestible carbohydrates, indole derivatives arise from microbial transformation of dietary tryptophan (105, 106). This gives them a different position in the therapeutic model, since their production depends not only on substrate availability, but also on the presence of microbial pathways capable of converting tryptophan into bioactive ligands (105, 106). Among these metabolites, indole-3-lactic acid (ILA) and indole-3-acetic acid (IAA) have attracted particular interest because they can influence epithelial and immune responses through AhR signaling (105, 106).

Recent preclinical data suggest that ILA and IAA may attenuate CIA through AhR-dependent mechanisms (107, 108). In this model, treatment with these metabolites was associated with reduced arthritis severity and increased regulatory T cell responses (107, 108). In parallel, ILA improved epithelial barrier properties in Caco-2 cells, with increased expression of zonula occludens-1 (ZO-1) nd occludin. The use of the AhR antagonist CH223191 abolished this effect, supporting the involvement of AhR signaling in this experimental model (107, 108).

These observations are relevant because they place tryptophan metabolism close to the interface between diet, microbiota and epithelial barrier function (109, 110) and suggest that metabolite-based approaches may not be limited to increasing SCFA production (109). A diet that provides tryptophan substrate, or a microbiota capable of producing AhR-active indoles, may influence mucosal homeostasis in ways that differ from fiber fermentation (105, 106, 109). However, these two metabolite axes should be regarded as complementary rather than interchangeable (109, 111).

The translational interpretation must remain cautious as the available evidence for ILA and IAA in arthritis is still preclinical, and the therapeutic significance of these metabolites in human RA remains undetermined (107). Human studies have reported alterations in tryptophan-related metabolic pathways in RA, but such findings are associative and do not show that supplementation with a specific indole metabolite improves disease activity or restores barrier function (112–114).

Future studies should determine whether patients with RA differ in their capacity to generate AhR-active indole metabolites, whether this capacity is related to intestinal permeability or disease activity, and whether it can be modified safely through diet, prebiotics, probiotics, or direct metabolite delivery.

### 3.5 Postbiotics and bacterial extracellular vesicles

The ISAPP consensus defines a postbiotic as a preparation of inanimate microorganisms and/or their components that confers a health benefit on the host (115, 116). Such preparations may contain microbial metabolites, but purified metabolites alone do not meet this definition. Butyrate and tryptophan-derived indoles are therefore discussed separately here as microbial effector molecules, although their proposed effects on epithelial and immune function overlap with those investigated for postbiotic preparations in RA.

The therapeutic appeal of postbiotics lies partly in their difference from live probiotics. Inactivated preparations do not require colonization, are generally more stable during storage and may reduce some risks associated with administering viable microorganisms to vulnerable patients (117, 118). This is relevant to autoimmune disease, where patients may receive glucocorticoids, biologic or targeted synthetic DMARDs (7, 119). However, the absence of viability does not by itself prove safety or efficacy. A postbiotic preparation still requires strain-level identification, toxicological assessment, dose definition, and controlled clinical testing in the intended population (115, 116).

In RA, the rationale for postbiotic approaches is still largely extrapolated from experimental and gastrointestinal studies (120, 121). Proposed benefits include support of epithelial barrier integrity, modulation of mucosal immune responses, reduction of inflammatory signaling, and effects on bone-related pathways (120, 122, 123).

Bacterial extracellular vesicles (EVs) carry proteins, lipids, nucleic acids, polysaccharides and metabolites that may interact with epithelial and immune cells without requiring colonization by live bacteria (124, 125). Their relevance as microbial effectors can therefore be considered alongside postbiotics, although classification of an EV preparation as a postbiotic requires assessment against the ISAPP criteria, including evidence of a health benefit in the intended host (116).

Experimental studies suggest that probiotic-derived EVs may influence inflammatory signaling, macrophage polarization, epithelial responses, and osteoclast-related pathways (126). These observations are relevant to RA because they point to cell-free microbial products as possible modulators of inflammation and barrier function (126). However, most of this evidence remains preclinical, and direct clinical evidence in patients with RA is currently lacking (126).

For bacterial EVs to be evaluated as a treatment for RA, their composition, purity and biological potency need to be reproducible across preparations. This requires consistent methods of production and storage (127, 128). The dose and route of administration also remain to be established. For oral delivery, vesicle stability and uptake at the intestinal mucosa need to be assessed, together with the extent of systemic exposure (129, 130). Safety assessment needs to consider both contaminants remaining in the preparation and unwanted immune responses to the vesicle components themselves, particularly in patients receiving immunosuppressive treatment (124, 127). These data on preparation quality and safety would support regulatory assessment and the design of controlled trials in RA, in which clinical outcomes are measured alongside intestinal and immune responses (127, 130, 131).

### 3.6 Nutrients with barrier relevance: supportive but not RA-specific therapy

Several nutrients, including amino acids, trace elements, fat-soluble vitamins, and bioactive plant compounds have been shown to support the integrity of the intestinal barrier. These factors should be considered, as they may influence epithelial metabolism, the regulation of TJs, mucosal immune functions, or the metabolic activity of microorganisms (132, 133). However, their therapeutic significance in RA requires careful interpretation as most evidence comes from experimental studies or other disease states rather than from controlled trials designed to assess barrier repair in patients with RA (60, 131).

Glutamine is one of the best studied nutrients in relation to intestinal epithelial function (134). It is a conditionally essential amino acid during metabolic stress and represents an important substrate for enterocytes and immune cells (134). In experimental epithelial systems, glutamine deprivation can impair barrier function and alter the expression or distribution of TJ proteins, while glutamine supplementation may partially restore epithelial resistance under selected injurious conditions (134). Although these observations provide a plausible biological rationale for considering glutamine in barrier-supportive nutrition, clinical data are not specific to RA and remain heterogeneous across gastrointestinal and inflammatory conditions (60, 134).

Zinc is another nutrient of potential relevance to epithelial integrity (134–136). It contributes to cellular proliferation, antioxidant defense, immune regulation, and the maintenance of epithelial junctional complexes (134–136). Zinc deficiency has been associated with impaired intestinal barrier function in experimental systems and may increase epithelial vulnerability to inflammatory stress (134–138). In clinical practice, this supports correction of zinc deficiency where present, particularly in patients with poor nutritional intake, chronic gastrointestinal symptoms, or other risk factors for micronutrient depletion (131). However, routine zinc supplementation as a gut-targeted intervention in RA is not supported by direct clinical evidence (60, 131, 135). Excessive zinc intake may also interfere with copper metabolism, and supplementation should therefore be approached as nutritional correction rather than empirical immunomodulation (135, 136).

Vitamin D has relevance for both immune regulation and skeletal health in RA, and has also been linked with the maintenance of intestinal barrier function. The vitamin D receptor is expressed by intestinal epithelial cells and by several immune cell populations, including T cells, B cells, dendritic cells, and macrophages. Experimental work has suggested that vitamin D-dependent mechanisms may support epithelial barrier integrity by influencing tight-junction proteins, antimicrobial peptide production, mucosal immune activity, and the composition of the gut microbiota (135, 139, 140). In patients with RA, assessment and correction of vitamin D deficiency is clinically important, particularly in relation to bone health, glucocorticoid treatment, and fracture risk (139, 140). However, this should not be taken to imply that vitamin D supplementation has been established as a specific treatment aimed at restoring gut barrier function in RA (140).

Polyphenols are widely present in plant-based foods, including fruits, vegetables, tea, cocoa, legumes, herbs, and olive oil (141). They are of particular interest in the context of the gut–joint axis because many polyphenols are poorly absorbed in their original form and are instead metabolized by intestinal microorganisms into smaller bioactive compounds (54, 142). Through these processes, they may modify microbial composition, epithelial redox status, and inflammatory signaling (142). However, in RA, the strongest evidence remains related to whole dietary patterns, especially Mediterranean-style diets, rather than to supplementation with isolated polyphenols (142). This is an important distinction, since such dietary patterns also provide fiber, unsaturated fatty acids, minerals, vitamins, and numerous other bioactive compounds, making it difficult to ascribe any clinical effect to polyphenols alone (143).

## 4 Live microbiota-based interventions: probiotics, live biotherapeutics and fecal microbiota transplantation

### 4.1 Probiotics and defined commensals

Probiotics and defined commensal organisms represent a form of gut-directed intervention that differs from diet, prebiotics or direct metabolite administration (144, 145). Instead of providing substrates for microbial metabolism, or delivering a microbial metabolite in a defined form, these approaches involve the administration of live microorganisms with the aim of altering microbial activity, epithelial responses or mucosal immune regulation (144, 145). However, the effects of such interventions should not be considered as general properties of probiotics as a category, since they depend on the strain used, dose, formulation, timing of administration, background diet, resident microbiota and disease status of the host (144–146). **Table 3** summarizes the weight of evidence for probiotic, defined commensal, and FMT-based interventions in RA, highlighting the distinction between clinical and mechanistic findings and the strain-specific nature of observed effects.

**Table 3:** Microbiota-based interventions: probiotics, defined commensals, and FMT.

Intervention	Key finding/claim	Weight of evidence	Studies (supporting or refuting)	References
*Lactobacillus rhamnosus* GG	A small pilot trial did not demonstrate significant improvement in RA activity compared with placebo.	Moderate	Hatakka et al. (2003): Double-blind, placebo-controlled pilot RCT; 26 randomized and 21 completed; *Lactobacillus rhamnosus* GG (LGG) (ATCC 53103), two capsules twice daily, each containing ≥5 × 10^9^ CFU (≥2 × 10¹^0^ CFU/day), for 12 months. Antirheumatic medication had been stable for at least 3 months; NSAIDs and low-dose corticosteroids were used, while DMARDs and prednisone doses >7.5 mg/day were exclusion criteria. No significant between-group improvement in clinical disease activity or HAQ was demonstrated. Fecal recovery of LGG was assessed for adherence, and fecal urease activity was measured without a significant between-group difference. Functional intestinal permeability tests and barrier biomarkers were not reported.	(150)
*L. rhamnosus* GR-1 and *L. reuteri* RC-14	Clinical benefit was not demonstrated; within-group functional improvement should not be interpreted as superiority over placebo.	Moderate	Pineda et al. (2011): Double-blind, placebo-controlled pilot RCT; 29 participants; 2 × 10^9^ CFU/capsule twice daily for 3 months, alongside stable RA medication. ACR20 responses occurred in 20% versus 7% of participants, without a significant between-group difference. HAQ improved within the probiotic group, but not significantly versus placebo. No microbiome or functional permeability outcomes were reported.	(151)
*Bacillus coagulans* GBI-30, 6086-containing preparation	Selected symptomatic improvements were reported, but the contribution of the probiotic cannot be separated from that of other active ingredients.	Moderate	Mandel et al. (2010): Double-blind, placebo-controlled pilot RCT; 45 randomized and 44 analyzed; 2 × 10^9^ CFU/day for 60 days. Usual RA treatment continued, including DMARDs in most participants. The preparation also contained green tea extract, methylsulfonylmethane, vitamins and minerals. Selected pain and functional outcomes improved. No microbiome or functional permeability outcomes were reported.	(292)
*L. casei* 01	Short-term clinical and inflammatory changes support further investigation of this preparation, but do not establish intestinal mediation or a general probiotic effect.	Moderate	Alipour et al. (2014) and Vaghef-Mehrabany et al. (2014): Complementary reports from one double-blind, placebo-controlled RCT; 60 randomized and 46 analyzed; ≥10^8^ CFU/day for 8 weeks alongside stable background RA treatment. Improvements in DAS28, tender and swollen joint counts and hs-CRP were reported, together with changes in selected circulating cytokines. No microbiome or functional permeability outcomes were reported. These publications should not be counted as independent trials.	(147, 148)
*L. acidophilus*, *L. casei* and *Bifidobacterium bifidum* preparation	Reported clinical improvement requires particular caution because an Expression of Concern has been issued.	Not assigned‡	Zamani et al. (2016): Double-blind, placebo-controlled RCT; 60 participants; one capsule/day for 8 weeks containing *L. acidophilus*, *L. casei* and *B. bifidum*, each at 2 × 10^9^ CFU/g. Strain identifiers and capsule content mass were not reported; total daily CFU could therefore not be determined. Background treatment included methotrexate, prednisolone and hydroxychloroquine; sulfasalazine was also listed in the methods. Individual medication doses were not reported. Reductions in DAS28 and hs-CRP were reported. Microbiota composition, functional intestinal permeability tests and barrier biomarkers were not reported; fecal SCFAs were explicitly not measured. An Expression of Concern has subsequently been issued, and the findings should not be treated as confirmatory evidence.	(153) (154);
Five-strain probiotic mixture	Changes in selected systemic inflammatory markers occurred without significant improvement in DAS28.	Moderate	Cannarella et al. (2021): Double-blind, placebo-controlled RCT; 47 randomized and 42 completed; one 2-g sachet/day for 60 days containing *L. acidophilus* La-14, *L. casei* Lc-11, *Lactococcus lactis* Ll-23, *B. lactis* Bl-04 and *B. bifidum* Bb-06, each at 10^9^ CFU/g (calculated total: 10¹^0^ CFU/day). RA therapy had been unchanged for at least 4 months and was maintained during the study. Methotrexate use was more frequent in the probiotic group (81% vs 48%), whereas prednisone use was more frequent in the placebo group (71% vs 33%). TNF-α and IL-6 changes favored probiotics, but DAS28, hs-CRP and ESR did not differ significantly between groups. Gut microbiota was not assessed; functional intestinal permeability tests and barrier biomarkers were not reported.	(152)
*L. casei* BLn2401, *L. salivarius* BL2201 and *B. breve* BL3406	More favorable clinical changes were reported in newly diagnosed RA, but independent confirmation and evidence of intestinal mediation are needed.	Moderate	Barac et al. (2025): Single-center, double-blind, placebo-controlled RCT; 100 participants; 5 × 10^9^ CFU/day for 12 months. Participants had recently started conventional treatment, including MTX and/or antimalarials and/or corticosteroids. More favorable longitudinal changes in DAS28 and selected clinical outcomes were reported with probiotics. No microbiome or functional permeability outcomes were reported.	(149)
*L. casei* CCFM1074	Experimental benefits are strain- and model-specific and cannot be extrapolated to *L. casei* 01 in patients with RA.	Emerging	Fan et al. (2021): Oral administration in CIA rats reduced arthritis severity and joint damage, with changes in Treg/Th17 balance, gut microbial composition and fecal propionate and butyrate concentrations. These findings do not establish the mechanism or clinical efficacy of a different *L. casei* strain in humans.	(156)
*Prevotella histicola*	A defined commensal suppressed experimental arthritis, with accompanying mucosal immune and barrier-related changes.	Emerging	Marietta et al. (2016): Oral treatment in HLA-DQ8 humanized mice reduced arthritis incidence and severity. Findings included changes in mucosal immune responses and barrier-related measurements, with decreased Th17 cells and increased regulatory T cells in mesenteric lymph nodes. Clinical efficacy in RA has not been established.	(158)
*Bacteroides fragilis* and propionate	Microbial metabolite-dependent protection was demonstrated experimentally; clinical efficacy remains unproven.	Emerging	Chen et al. (2024): Microbiota transfer from CIA-resistant mice conferred protection, and *B. fragilis* was identified as a protective organism. Propionate modulated HDAC3–FOXK1 and IRF3/IFN-β signaling in RA-FLS. Propionate combined with etanercept enhanced suppression of experimental arthritis. These findings do not establish a treatment combination for human RA.	(161)
*Peptoniphilus gorbachii*	Human serological associations and experimental protection provide different levels of evidence; treatment effects remain preclinical.	Emerging	Kim et al. (2023): A human observational component included 81 RA patients and 50 controls; lower serum IgM reactivity to *P. gorbachii* was associated with greater disease activity. In CIA mice, oral administration reduced arthritis severity and altered intestinal inflammatory responses and tight-junction proteins. Cecal SCFA concentrations did not differ significantly, and mediation by butyrate was not established. Human antibody measurements do not demonstrate intestinal abundance or a protective effect in patients.	(160)
Fecal microbiota transplantation	RA-specific clinical evidence remains limited to case-level observations.	Emerging	Zeng et al. (2021): Single RA case report. Improvement in RA symptoms was reported after FMT. An uncontrolled observation cannot distinguish the intervention’s effect from background treatment, disease fluctuation or other confounders and does not establish efficacy or an intestinal mechanism.	(168)

Weight of evidence is a descriptive assessment of the evidence supporting the stated finding or claim, taking into account study design, methodological limitations, consistency and relevance to RA. Strong denotes consistent evidence from adequately conducted human studies; Moderate denotes human evidence limited by study size, design or inconsistent findings; Emerging denotes preliminary evidence from experimental models, case reports or conference abstracts. Indirect identifies evidence from human populations without RA that is extrapolated to RA. These categories do not constitute a formal GRADE assessment. The rating applies to the outcome described: evidence of clinical benefit does not establish mediation through the gut microbiota or intestinal barrier, and changes in intestinal or circulating biomarkers do not by themselves demonstrate clinical efficacy.

^‡^The Zamani study is retained for transparency but is not assigned an evidence rating because an Expression of Concern has been issued.

ACR20, American College of Rheumatology 20% response criteria; CFU, colony-forming units; CIA, collagen-induced arthritis; CRP, C-reactive protein; DAS28, Disease Activity Score in 28 joints; DMARDs, disease-modifying antirheumatic drugs; ESR, erythrocyte sedimentation rate; FMT, fecal microbiota transplantation; HAQ, Health Assessment Questionnaire; hs-CRP, high-sensitivity CRP; MTX, methotrexate; RA, rheumatoid arthritis; RA-FLS, rheumatoid arthritis fibroblast-like synoviocytes; RCT, randomized controlled trial; SCFAs, short-chain fatty acids; Th17, T helper 17 cells; Tregs, regulatory T cells.

Human trials of probiotic supplementation in RA have produced mixed results. In an eight-week placebo-controlled trial of Lactobacillus casei 01 at ≥10^8^ CFU/day, analysis of 46 participants showed an improvement in DAS28 (147, 148). More favorable clinical outcomes were also reported in a trial involving patients with newly diagnosed RA receiving conventional treatment (149). However, other trials did not demonstrate significant between-group improvements in disease activity or clinical response. These included the studies by Hatakka et al. and Pineda et al., which assessed disease activity and ACR20 response, respectively (150, 151). Cannarella et al. reported changes in selected inflammatory markers without a significant between-group difference in DAS28 (152). The positive findings reported by Zamani et al. should be interpreted cautiously because the journal issued an Expression of Concern over research integrity and reporting discrepancies, with doubts remaining in the absence of verification of clinical records (153, 154). Differences in probiotic preparations, treatment duration and background therapy limit comparisons between these studies. **Table 3** provides the study characteristics, clinical outcomes and available intestinal measurements.

Pooled evidence also requires cautious interpretation. In the RA analysis of the broader systematic review by Zeng et al., four trials contributed to the DAS28 meta-analysis, which found no statistically significant difference between intervention and control groups (weighted mean difference −0.55, 95% CI −1.33 to 0.24; I² = 97%). A pooled reduction in CRP was reported, but this should not be equated with improved disease activity (155). The inclusion of the Zamani et al. trial, subsequently subject to an Expression of Concern, adds uncertainty to the interpretation of this meta-analysis (153–155).

The human trials summarized in **Table 3** do not establish that changes in the microbiota or intestinal barrier mediated clinical responses. Functional intestinal permeability outcomes were not reported, and the available intestinal measurements do not demonstrate such mediation. Taken together, these findings support further evaluation of defined preparations but do not establish a consistent clinical benefit or a gut-mediated mechanism sufficient to support routine probiotic supplementation as an adjunct to RA treatment.Studies in experimental arthritis models have identified several defined bacteria that can reduce disease severity, although the effects vary with the organism and experimental conditions. In rats with CIA, *Lactobacillus casei* CCFM1074 altered the Treg/Th17 balance, microbiota composition and metabolite profiles, whereas the protection observed with *Bifidobacterium adolescentis* depended on when it was administered (156, 157). *Prevotella histicola*, a commensal isolated from the human gut, suppressed arthritis in humanized mice. This effect was accompanied by changes in mucosal immune responses and measurements related to intestinal barrier function (158, 159). In mice with CIA, *Peptoniphilus gorbachii* reduced arthritis scores and increased intestinal tight-junction proteins while protection associated with *Bacteroides fragilis* was linked to propionate production (160, 161). These bacteria are candidates for further investigation, but the findings remain specific to the experimental settings in which they were studied and do not establish efficacy in patients with RA.

Human data are still limited and mainly concern selected *Lactobacillus*-containing preparations, whereas much of the strain-specific evidence comes from CIA or humanized mouse models (147, 151, 156–160, 162, 163). Future studies should use well-characterized strains, report exact taxonomy and dose, assess persistence or colonization where relevant, and include microbiome, metabolite, barrier, and immune readouts together with standard RA outcomes (145, 164).

### 4.2 Fecal microbiota transplantation

Fecal microbiota transplantation represents the most complex form of microbiota-based intervention (165). In contrast to a probiotic strain or a defined microbial metabolite, FMT transfers a whole microbial community along with other stool-derived biological components, including phages and microbial products (165, 166). This broad biological composition makes the intervention difficult to standardize (166, 167). The donor, the recipient, the route of administration, pretreatment, formulation, storage conditions, and baseline microbiota may all influence the final effect (165–167).

Clinical evidence for FMT in RA remains very limited. To date, the RA-specific clinical literature consists mainly of a single case report describing improvement in RA symptoms after FMT (168). However, it cannot be used to infer efficacy as a case report does not control for background treatment, fluctuation of disease activity, placebo effects, diet, infection history, or other environmental exposures. It should therefore be treated as a reason for controlled studies, not as support for clinical use (168, 169).

Evidence from other types of inflammatory arthritis also supports exercising caution. The FLORA trial examined FMT in active peripheral psoriatic arthritis using an exploratory, double-blind, sham-controlled randomized design (170). This study did not show clinical benefit of FMT over sham treatment, and some outcomes favored the sham group (170). Although psoriatic arthritis is not RA, these findings are relevant as a cautionary example, showing that transfer of a donor microbial community does not automatically translate into clinical improvement in inflammatory joint disease. They also indicate that donor selection, recipient biology, and safety assessment cannot be treated as secondary issues (170). A subsequent analysis of the FLORA cohort suggested that the baseline microbial community of the recipient may influence donor strain engraftment and longer-term response (170–172). A follow-up analysis of the FLORA trial showed that FMT, compared with sham transplantation, altered the dynamics of 12 inflammation-associated plasma proteins in patients with psoriatic arthritis, including IFN-γ; however, because IFN-γ was already elevated in PsA patients relative to healthy controls and was further increased after FMT, the clinical significance of this change remains uncertain and requires prospective validation (173).

Several technical and clinical problems also limit the translation of FMT to RA. Donor screening must exclude transmissible pathogens and reduce the risk of transferring undesirable metabolic, inflammatory or antimicrobial-resistance traits (174). Capsule-based formulations may differ from fresh or frozen preparations, and processing may alter both viable and non-viable components of the microbial product. Repeated dosing, route of administration, durability of engraftment, and long-term safety remain uncertain in autoimmune rheumatic diseases (175). These issues are particularly relevant in patients receiving glucocorticoids, biologic or targeted synthetic DMARDs (174). Taken together, FMT remains an experimental microbiota-based intervention and is not supported for routine treatment of patients with RA.

## 5 Role of physical exercise, intestinal homeostasis and the gut–joint axis in RA

Exercise is an established adjunct to pharmacological treatment in RA, and its clinical use is addressed in EULAR and ACR recommendations (175–177). These recommendations provide the background for considering a less well understood question: whether intestinal changes induced by exercise contribute to clinical outcomes in RA. Improvements in physical function and symptoms do not, on their own, establish a role for the gut microbiota or intestinal barrier.

### 5.1 Clinical benefits of exercise in RA

Aerobic and resistance exercise have complementary effects on cardiorespiratory fitness, muscle strength and physical function in patients with RA (178, 179). Benefits for pain, fatigue and quality of life have also been reported, although findings vary with the population studied and the training program (180–182). The clinical rationale for exercise therefore does not depend on evidence of an intestinal mechanism.

Exercise programs need to take into account disease activity, joint damage, comorbidities and the patient’s initial capacity, with gradual progression and adjustments in response to symptoms or functional limitations (175–177). A detailed prescription is outside the scope of this Review. Here, we examine whether exercise-related changes in microbial function, intestinal barrier measures or immune responses may help explain clinical benefits in RA, or merely occur alongside them. **Table 4** summarizes the established clinical effects and distinguishes them from findings that provide indirect support for a gut-mediated response.

**Table 4:** Role of physical exercise in rheumatoid arthritis.

Intervention	Key finding/claim	Weight of evidence	Studies (supporting or refuting)	References
Aerobic and Resistance Exercise as Structured Adjuncts to Pharmacological RA Treatment	Exercise is recommended as a structured adjunct to pharmacological RA treatment. Aerobic training improves cardiorespiratory fitness and pain; resistance training targets muscle strength and lean mass. EULAR (2025 update) and ACR guidelines endorse exercise adapted to disease activity and individual capacity, following the FITT framework (frequency, intensity, time, type). Exercise should not be withheld from patients with controlled disease activity.	Strong	Zhang et al. (2025) Network meta-analysis of 21 RCTs (n=1,520): traditional Chinese exercise and yoga significantly improved DAS28-ESR vs usual care. SUCRA analysis indicated TCE as the highest-probability optimal modality. No exercise modality demonstrated significant CRP or ESR reduction.	(238)
			Ye et al. (2022) Aerobic exercise systematic review and meta-analysis (13 RCTs, n=967). Significantly improved functional ability (MD=−0.25; 95%CI −0.38 to −0.11; moderate certainty) and pain (SMD=−0.46; p=0.04); aerobic capacity improved (MD =+ 2.41 mL/kg/min). CRP and ESR effects were low certainty due to between-study inconsistency.	(293)
			Hu et al. (2021) Overview of systematic reviews: confirmed improvements in aerobic capacity, muscle strength, functional ability, and fatigue across multiple RCT cohorts. Combined aerobic and resistance programs showed additive cardiometabolic benefit.	(294)
Exercise-Induced Modulation of Gut Microbiota Composition, Diversity, and SCFA-Producing Bacteria in Non-RA Populations	Exercise may support SCFA-producing bacterial taxa and microbiota diversity in some settings, but direct evidence in RA is absent. More than half of human intervention studies show no clear change in alpha-diversity after exercise training.	Indirect	Cullen et al. (2023) Systematic review: in the included human studies, exercise was generally associated with a decrease in the *Bacillota/Bacteroidota* ratio and consistent increases in *Bacteroides* and *Roseburia*. However, heterogeneous populations and exercise protocols limit generalization of these findings. Changes in this ratio should not be interpreted as evidence of improved intestinal barrier function or clinical benefit in RA. SCFAs were the most frequently measured microbial metabolites, but the limited available studies showed no clear pattern of exercise-related changes.	(209)
			Min et al. (2024) Meta-analysis of 25 exercise intervention studies (1,044 participants): exercise significantly affects beta diversity but not alpha diversity consistently. Enrichment of *Akkermansia muciniphila* and butyrate-producing clusters (*Roseburia, Faecalibacterium prausnitzii*) observed in several studies.	(211)
			Hintikka et al. (2023) Multi-omic study in 17 women with overweight, involving a 6-week control period followed by 6 weeks of endurance exercise. Training was associated with changes in serum and fecal metabolomic profiles, including increases in serum lysophosphatidylcholines and fecal glycerophosphocholine. This metabolic signature was associated with *Akkermansia* abundance and microbial metagenomic pathways. These findings suggest exercise-related interactions between host metabolism and the gut microbiome, but do not establish increased circulating butyrate, improved intestinal barrier function, or clinical benefit in RA.	(223)
			Reljic et al. (2025) Pooled analysis of randomized trials in patients with obesity and metabolic syndrome: fecal SCFAs increased in the high-intensity interval-training group, but not in the moderate-intensity group. Both exercise programs included resistance training. These findings concern a non-RA population and do not establish intestinal mediation of clinical benefit in RA.	(216)
Immunometabolic effects of exercise	Exercise may influence inflammatory markers and monocyte activation, although their relationship to intestinal changes in RA remains uncertain. Reviews and pilot RA studies suggest effects on immune cells and inflammatory markers, but findings are heterogeneous.	Moderate	Bartlett et al. (2018) Uncontrolled pilot study in 12 patients with stable RA:10-week high-intensity interval walking significantly reduced CD14^+^CD16^+^ monocyte proportion, lowered TLR2/TLR4/HLA-DR on intermediate monocytes, and improved E. coli phagocytosis. DAS28 and aerobic fitness improved; no consistent changes in IL-6, CRP, TNF-α, or IL-1β - suggesting that exercise-related cellular immune changes may occur without parallel changes in circulating inflammatory mediators, although the biological significance of this pattern remains uncertain.	(203)
			Modarresi Chahardehi et al. (2022) Systematic review: variable CRP and ESR reductions across RA exercise studies; most consistent anti-inflammatory effects seen with aerobic modalities; myokines, adipokines, and redox signaling identified as candidate mechanisms linking muscle activity to systemic immune modulation.	(188)

Weight of evidence is a descriptive assessment of the evidence supporting the stated finding or claim, taking into account study design, methodological limitations, consistency and relevance to RA. Strong denotes consistent evidence from adequately conducted human studies; Moderate denotes human evidence limited by study size, design or inconsistent findings; Emerging denotes preliminary evidence from experimental models, case reports or conference abstracts. Indirect identifies evidence from human populations without RA that is extrapolated to RA. These categories do not constitute a formal GRADE assessment. The rating applies to the outcome described: evidence of clinical benefit does not establish mediation through the gut microbiota or intestinal barrier, and changes in intestinal or circulating biomarkers do not by themselves demonstrate clinical efficacy.

ACR – American College of Rheumatology; CAT – catalase; CD14^+^CD16^+^ monocytes – monocyte subset expressing CD14 and CD16; CI – confidence interval; CRP – C-reactive protein; DAS28 – Disease Activity Score in 28 joints; DAS28-ESR – Disease Activity Score based on 28 joints and erythrocyte sedimentation rate; *E. coli* – Escherichia coli; ESR – erythrocyte sedimentation rate; EULAR – European Alliance of Associations for Rheumatology; FITT – frequency, intensity, time, and type, a framework used for exercise prescription; GPx – glutathione peroxidase; HLA-DR – human leukocyte antigen DR, a marker of antigen-presenting cell activation; IL-1β – interleukin-1 beta; IL-6 – interleukin-6; MD – mean difference; MDA – malondialdehyde, a marker of lipid peroxidation; mL/kg/min – milliliters of oxygen per kilogram of body mass per minute, commonly used to express aerobic capacity; RA – rheumatoid arthritis; RCT – randomized controlled trial; SCFAs – short-chain fatty acids; SMD – standardized mean difference; SOD – superoxide dismutase; SUCRA – surface under the cumulative ranking curve, a metric used in network meta-analysis to rank interventions; TCE – traditional Chinese exercise; TLR2/TLR4 – Toll-like receptor 2/Toll-like receptor 4; TNF-α – tumor necrosis factor alpha.

### 5.2 Rheumatoid cachexia, muscle function and metabolic health

Rheumatoid cachexia is characterized by loss of skeletal muscle, often in the presence of preserved or increased fat mass. Body weight alone can therefore underestimate its functional consequences (183, 184). Resistance training improves muscle strength and may increase lean mass, whereas programs combining aerobic and resistance exercise address complementary aspects of functional and metabolic health (179, 185–187).

These effects also need to be considered when interpreting the intestinal findings of exercise studies. Improvements in muscle function, fitness or body composition may contribute to a clinical response without requiring changes in the gut. Measuring these outcomes alongside microbial and barrier-related variables allows the different responses to be examined, but their occurrence together does not establish intestinal mediation. The following subsection examines the proposed immunometabolic and intestinal pathways, with attention to the evidence available in RA.

### 5.3 Anti-inflammatory, immunometabolic and intestinal effects of exercise

Physical exercise in RA may also influence the inflammatory and metabolic environment in which the disease develops and persists (188). Several studies have reported reductions in systemic inflammatory markers after exercise interventions in RA, including changes in CRP, ESR, and selected cytokines (188, 189). These findings are not uniform, and the magnitude of change is likely to depend on disease activity, medication, body composition, baseline fitness, and the type of training used (188, 190).

Potential mechanisms linking exercise with intestinal homeostasis include changes in gut motility, bile acid metabolism, microbial substrate availability, mucus layer function, antioxidant responses, and regulation of host–immune interactions. These pathways remain incompletely characterized in RA, but they provide a biological rationale for studying exercise as a modifier of the gut–joint axis rather than only as a functional intervention (191).

Skeletal muscle may contribute to these effects through its endocrine activity (192). During contraction, muscle releases myokines and other soluble mediators that can influence energy metabolism, vascular function, and immune regulation (192). In RA, this could provide a plausible link between muscle activity and systemic inflammation (192, 193).

Exercise also influences the number, distribution, and functional state of circulating immune cells (194, 195). A single bout of activity induces a rapid, but transient leukocytosis, with particularly marked changes in neutrophils, monocytes, natural killer cells, and differentiated T cell subsets (196). This response reflects hemodynamic shear stress, catecholamine-mediated demargination, and altered trafficking between the blood, vascular endothelium, and peripheral tissues, rather than a uniform activation of the immune system (194–196). After exercise, lymphocyte counts may temporarily fall below baseline, whereas neutrophils often remain elevated for longer, probably as a consequence of delayed mobilisation from marginated and bone marrow pools (194, 195).

Repeated training may further modify this response by altering the relative abundance and activation profile of innate immune cell populations (197). In inflammatory and metabolic diseases, regular exercise has been associated with a lower proportion of CD14^+^CD16^+^ inflammatory monocytes, reduced expression of Toll-like receptors (TLRs) and HLA-DR on selected monocyte subsets, and changes in phagocytosis, chemotaxis, and oxidative burst activity (197, 198). These observations are of particular interest in RA, since monocytes contribute to synovial macrophage replenishment, antigen presentation, cytokine production, and osteoclastogenic signaling, while neutrophils participate in synovial inflammation through degranulation, reactive oxygen species generation, protease release, and formation of neutrophil extracellular traps (199–202).

Lower expression of TLR2 and TLR4 could reduce the responsiveness of circulating monocytes to bacterial products entering the bloodstream from the intestine, potentially limiting the contribution of these cells to synovial inflammation (198, 199). This suggests a possible connection with the gut–joint axis, although it does not show that exercise reduces intestinal translocation or that this mechanism accounts for a reduction in joint inflammation.

The evidence directly obtained in RA is still limited, but suggests that exercise may influence innate immune cells, including monocytes, even when conventional circulating inflammatory markers do not change substantially (203). These observations come from an uncontrolled pilot study in which 12 older adults with stable RA completed ten weeks of interval walking; the absence of a comparison group limits attribution of the changes to exercise (203). In older adults with stable RA, a program of high-intensity interval walking reduced the relative burden of CD16-expressing inflammatory monocytes, TLR2, TLR4, and HLA-DR expression on intermediate monocytes, improved monocyte phagocytosis of *Escherichia coli*, and was accompanied by improved aerobic fitness and lower disease activity (203). These cellular changes occurred without consistent reductions in circulating IL-1β, IL-6, CXCL8, IL-10, TNF-α, or CRP (203). This suggests that exercise-related effects on innate immune cells may not always be reflected by parallel changes in circulating inflammatory mediators, although the biological significance of this dissociation remains uncertain (203).

Exercise has been proposed as a factor capable of modifying the gut microbiota; however, most data come from healthy adults, athletes, or overweight/obese individuals, and the vast majority of studies concern non-RA populations (204–206). These studies are useful because they show that the intestinal microbial community may respond to physical activity, but they do not demonstrate that exercise can correct RA-associated dysbiosis or alter the gut–joint axis in patients with established disease (13, 207, 208).

Systematic reviews indicate that the effect of exercise on microbial diversity is variable (209, 210). Some human studies have reported increases in alpha-diversity measures, including the Shannon index, which reflects both richness and evenness of the microbial community. However, more than half of human intervention studies have not shown clear changes in microbial diversity after exercise training (209). This means that diversity should not be treated as a uniform therapeutic endpoint. A higher Shannon index may indicate a broader microbial community in some settings, but its clinical meaning depends on the population studied, the sequencing method used, and the functional capacity of the microbiota (211, 212).

Changes in the *Bacillota/Bacteroidota* ratio, also described as the *Firmicutes/Bacteroidetes* ratio, vary across exercise studies and are influenced by participant characteristics and analytical methods; they should not be interpreted alone as evidence of a beneficial microbial response to exercise (209, 211, 213–215).

A more useful interpretation may come from microbial function. Several studies suggest that exercise may increase the abundance of taxa capable of producing SCFAs, or may alter the microbial pathways involved in energy metabolism and fermentation (209, 210, 216). However, the evidence remains inconsistent. In many studies, microbial metabolites were not measured directly, and taxonomic changes were used only as indirect indicators of metabolic function (216, 217). Therefore, it is more accurate to state that exercise may support SCFA-producing capacity in some settings, rather than that it reliably increases SCFA production.

In RA, this uncertainty is even greater. The effects of exercise on the gut microbiota in RA have not been established in well-controlled intervention studies (218–220). Rheumatoid arthritis also introduces additional sources of variation, including disease activity, glucocorticoid exposure, conventional and biologic DMARDs, analgesic use, diet, comorbidities, and reduced physical capacity. These factors may all influence the gut microbiota and should be recorded in future studies (16).

Some studies in populations without RA have linked physical exercise with greater gut microbial diversity, although this finding is not consistent across studies and does not establish a clinical benefit in RA (209, 221, 222). In a study involving overweight women, Hintikka et al. reported exercise-related changes in serum and fecal metabolites, including phospholipid-related changes associated with Akkermansia (223). Experimental evidence that butyrate can support epithelial barrier function and attenuate arthritis provides a biological rationale for investigating exercise-related changes in microbial metabolites (12, 224). However, these findings concern butyrate administration and do not demonstrate that exercise produces the same effects in patients with RA.

### 5.4 Exercise intensity, duration and intestinal permeability

Exercise may affect the intestinal barrier in different ways, depending on intensity, duration, and environmental conditions (225, 226). This distinction is important when physical activity is considered as supportive care in RA, since the term exercise may refer to repeated moderate training, prolonged endurance work or strenuous high-intensity effort (225). These forms of activity are not physiologically equivalent and should not be assumed to have the same effects on gut barrier function (226).

**Figure 2** summarizes proposed links between exercise and intestinal homeostasis, including adaptive responses to regular training and intestinal stress associated with prolonged exertion, heat and dehydration; their relevance depends on exercise intensity, duration and individual circumstances.

**Figure 2:**
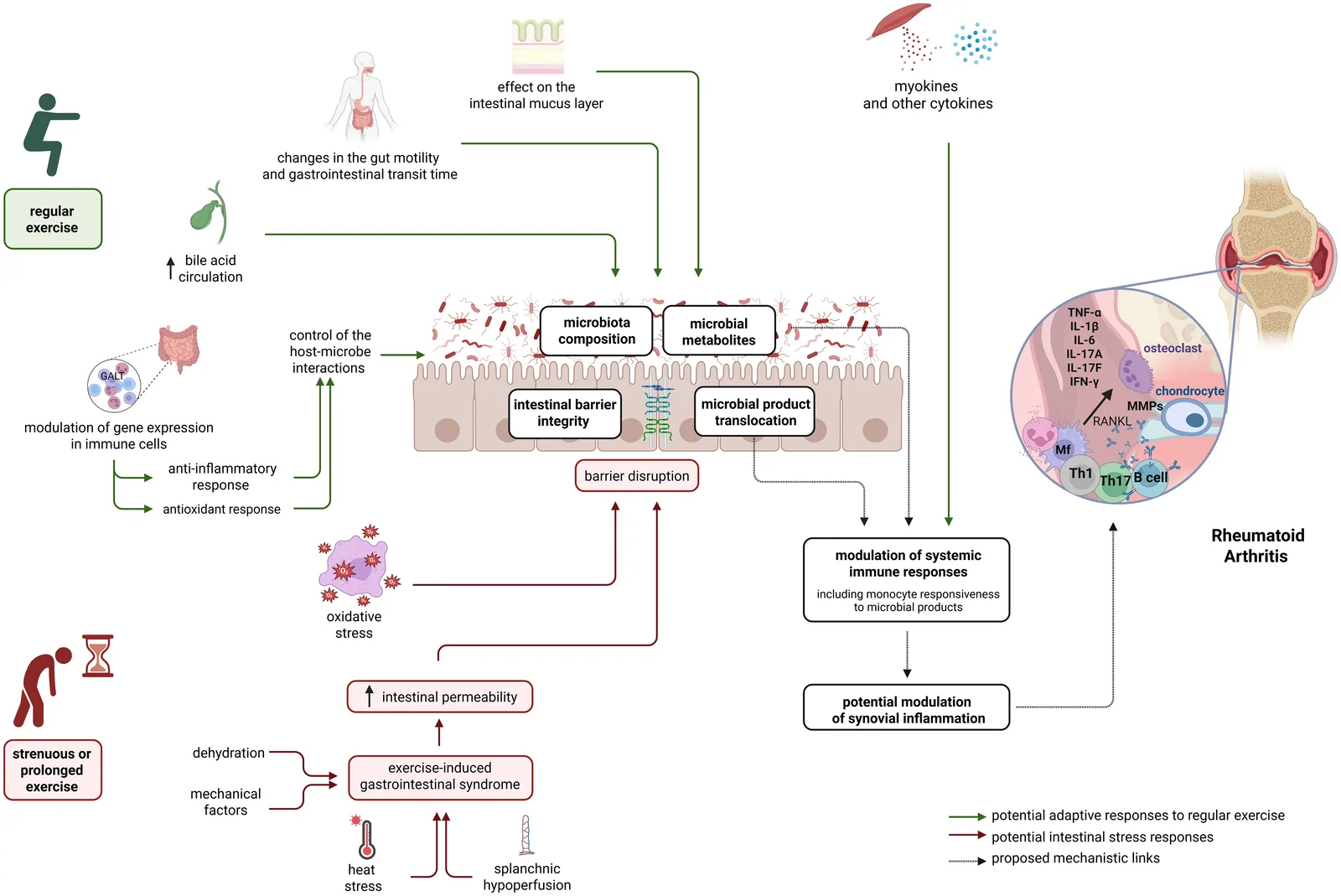
Exercise-related intestinal responses and their potential relevance to synovial inflammation in rheumatoid arthritis. Regular exercise may influence gut motility, the intestinal mucus layer, bile acid circulation and mucosal immune regulation, with possible effects on microbial composition, metabolite production and intestinal barrier integrity. Exercise-induced myokine and cytokine signaling may also modulate systemic immune responses. Strenuous or prolonged exercise, particularly in the presence of dehydration, heat stress and splanchnic hypoperfusion, may increase intestinal stress and permeability. Changes in microbial metabolites and exposure to microbial products could influence systemic immune responses, including monocyte responsiveness, and thereby affect synovial inflammation. Green arrows indicate potential adaptive responses to regular exercise; red arrows indicate potential intestinal stress responses; dotted arrows denote proposed mechanistic links. These links do not establish that intestinal changes mediate the clinical benefits of exercise in patients with RA. Likewise, intestinal stress responses to strenuous exercise should not be interpreted as evidence that exercise worsens RA through the gut. GALT, gut-associated lymphoid tissue; IFN-γ, interferon-γ; IL, interleukin; Mf, macrophage; MMPs, matrix metalloproteinases; RA, rheumatoid arthritis; RANKL, receptor activator of nuclear factor-κB ligand; Th, T helper cell; TNF-α, tumour necrosis factor-α. Created with BioRender.com.

Strenuous or prolonged exercise can induce a gastrointestinal stress response, often termed exercise-induced gastrointestinal syndrome (227). During intense exercise, blood flow is redistributed towards working skeletal muscle and skin, with a concomitant reduction in splanchnic perfusion. The intestinal epithelium may therefore be exposed to relative hypoperfusion and hypoxia. Heat stress, dehydration, and mechanical factors can further increase epithelial stress, particularly during prolonged endurance exercise or high-intensity exercise performed in hot conditions (227).

Strenuous or prolonged exercise has been associated with changes in markers of intestinal epithelial injury and barrier function, but these measurements describe different aspects of the intestinal response (228, 229). Increased intestinal fatty acid binding protein (I-FABP) indicates enterocyte injury and does not directly measure permeability, whereas the interpretation of zonulin-related measurements is limited by assay specificity (28, 29). Functional permeability tests and measurements of circulating microbial products also assess distinct processes. Changes in these measures should therefore not be taken as equivalent evidence of tight-junction disruption.

These acute responses should be distinguished from the effects of regular moderate exercise. Repeated low- to moderate-intensity training may improve metabolic health, reduce inflammatory tone and support adaptive responses in several tissues (222, 230–232). Experimental and clinical studies also suggest that moderate exercise may help maintain gut barrier integrity, although the evidence is not sufficient to define a single protective threshold for patients with RA (222, 226, 230–232). The final effect is therefore likely to depend on training status, disease activity, hydration, heat exposure, medication use and recovery (222).

A useful interpretation is provided by the hormetic model (230). According to this model, moderate repeated physiological stress may promote adaptation, whereas excessive stress may cause transient injury. Applied to the gut, moderate exercise may support protective responses and improve tolerance to later stress, while prolonged or very intense exercise may temporarily increase permeability, epithelial injury markers, and microbial product translocation (222, 230). This does not mean that exercise is harmful. It means that its biological effect depends on dose and context (222, 230).

For patients with RA, this distinction has practical importance. Regular moderate exercise should remain part of supportive care when adjusted to disease activity, joint status, comorbidity, and baseline fitness (233). By contrast, prolonged endurance exercise, high-intensity interval work, and training in heat should be introduced more cautiously, especially in patients with active disease, gastrointestinal symptoms, marked fatigue, poor conditioning, or medication use that may affect the gut (225). Future studies should combine exercise prescription with I-FABP, LPS-related markers, ZFPs, direct permeability tests and clinical outcomes, in order to determine whether gut barrier responses differ between RA patients and healthy controls (177, 234, 235).

## 6 Discussion

### 6.1 Translational interpretation of current evidence

The therapeutic relevance of gut–joint interactions depends on whether modifying the intestinal environment provides additional clinical benefit alongside established RA treatment (13, 236, 237) (91, 222).

As shown in **Tables 1**–**4**, the translational maturity of these interventions differs substantially, ranging from guideline-supported exercise and dietary approaches to preclinical barrier-, metabolite- and microbiota-directed strategies.

Pharmacological reinforcement of the epithelial barrier, dietary and prebiotic interventions, microbial metabolites, probiotics, postbiotic preparations, FMT, and physical activity may all influence one or more elements of the gut–joint axis (89, 121, 238, 239). These interventions may affect TJ regulation, epithelial cell survival, microbial metabolite production, antigen exposure, innate immune sensing, and the balance between inflammatory and regulatory immune responses (6, 91, 240). The strength of evidence supporting these approaches, however, is not uniform. In many cases, the most convincing mechanistic data have been obtained in experimental models of arthritis, whereas human studies are generally smaller, more heterogeneous, and often associative in character (8, 121, 169, 238, 239, 241).

Experimental data have provided evidence that impairment of the intestinal barrier or alteration of the gut microbiota can modify the severity of arthritis, supporting the biological plausibility of a gut–joint axis. However, in human RA, alterations in microbial composition and permeability-related biomarkers cannot yet be taken as evidence of causation (121, 238, 239). In some patients, such changes may occur before the onset of clinically apparent disease. In others, they may arise in parallel with systemic inflammation or reflect the effects of treatment, diet, comorbidity, or environmental exposure (121, 238, 239).

A similar degree of caution is required when considering therapeutic implications. Strategies aimed at improving intestinal barrier function or modifying microbial activity should not be regarded as replacements for established DMARDs (13). Their more plausible role is as adjunctive approaches that may help to maintain intestinal homeostasis, limit the passage of microbial products across the mucosa, support mucosal immune regulation, or modify treatment response in selected groups of patients (2, 13).

This point is especially important because patients with RA differ in clinical characteristics and in factors that may influence their response to gut-directed interventions (242). Patients differ in disease duration, autoantibody status, inflammatory activity, previous and current drug exposure, nutritional status, comorbidities, and baseline microbiota composition. These factors may substantially determine whether an intervention directed at the gut produces a measurable clinical or biological effect (242).

### 6.2 Patient stratification

Patient stratification is therefore likely to be an important condition for future clinical application (14, 236, 243, 244).

A major difficulty in therapies aimed at restoring intestinal permeability in RA is the marked heterogeneity of the disease. Patients vary in the degree of gut barrier dysfunction, dysbiosis and innate immune activation, which may explain why interventions directed at the gut barrier are likely to benefit only a defined subgroup. Selecting patients for treatment therefore requires biomarkers that can predict who is likely to respond. While increased ZFPs have been proposed for this purpose, their ability to predict a response to zonulin antagonists has yet to be validated (245, 246). Elevated ZFPs have also been observed in ACPA-positive individuals without arthritis and this finding warrants further study, but does not by itself show that ZFPs can predict progression to RA (246). In both settings, interpretation depends on the specificity of the assays used to measure zonulin-related proteins (28, 29).

Several candidate markers may be useful in research settings, although they should be grouped according to the biological process which they are intended to reflect. These markers describe different aspects of epithelial injury, barrier status and exposure to microbial products. For example, I-FABP reflects enterocyte injury, whereas occludin and claudin-1 measurements provide indirect information about the epithelial barrier (22, 247–249). A recent prospective controlled study reported lower baseline serum occludin and claudin-1 in active RA compared with controls. Greater baseline serum occludin and fecal claudin-1 were associated with a greater probability of remission after 6 months of anti-TNF therapy, while serum occludin and claudin-1 increased after treatment; however, these biomarkers should be interpreted as indirect markers of epithelial barrier status (22). Interpretation of ZFPs is complicated by the detection of proteins other than zonulin in some commercial assays, so these measurements cannot be taken as specific indicators of intestinal permeability (28, 29). LPS, LBP and sCD14 provide information about exposure to microbial products or the host response to them but they neither measure permeability directly nor establish that the microbial products originated in the intestine (247–250). Each measurement therefore needs to be interpreted according to what it assesses and, where possible, used alongside functional permeability tests (22).

Intestinal barrier abnormalities in patients with RA have been examined using several methods that provide different types of information. Tajik et al. (12) measured functional permeability through urinary recovery of lactulose and mannitol, reporting increased lactulose/mannitol ratios in small groups of patients with new-onset and established RA. Earlier studies measured the urinary recovery of orally administered ^5^¹Cr-EDTA. In the a study by Jenkins et al. (251), abnormal results were found predominantly in patients taking NSAIDs, indicating the need to consider medication exposure when interpreting these findings. Such functional tests differ from serum zonulin-related assays and measurements of epithelial injury markers or circulating tight-junction proteins, which provide indirect information about the barrier. Interpretation therefore depends on the population studied, its treatment exposure and the method used.

Microbial taxonomy also needs careful interpretation. Enrichment of selected taxa, including *Prevotellaceae, Segatella copri* (formerly *Prevotella copri*), *Collinsella, Eggerthella, Escherichia, Klebsiella*, or *Ruminococcus gnavus*, and depletion of butyrate-producing bacteria such as *Faecalibacterium, Roseburia, Coprococcus*, or other *Lachnospiraceae* and *Ruminococcaceae*, may be biologically informative (13, 17, 252–254). Taxonomic differences alone, however, are unlikely to provide a sufficient basis for clinical decisions. The same organism may have different effects depending on strain, host context and metabolic activity (13, 17, 242, 255).

This point is particularly relevant for *Segatella copri*, since genomic analysis have shown that the *P. copri* complex comprises multiple distinct clades with substantial functional diversity, including differences in carbohydrate metabolism (256). Therefore, the presence or abundance of *S. copri/P. copri* alone may be insufficient to infer pathogenic relevance, dietary responsiveness, or therapeutic implications without strain- or clade-level and functional characterization.

For this reason, strain-resolved metagenomics, functional profiling and longitudinal assessment of microbiota instability may provide more useful information than simple comparisons of bacterial abundance (8, 17, 237, 257). A patient with transient microbiological instability around the peri-onset phase may therefore represent a different therapeutic target than a patient with long-term, treated RA, in whom the microbiome may be shaped by chronic inflammation and medication exposure (17, 258).

Metabolomic profiling may be particularly informative, since many gut-directed interventions aim to restore microbial function rather than only change microbial composition (114, 191, 259). Short-chain fatty acids, especially butyrate and propionate, tryptophan-derived indole metabolites, bile acids, polyamines, and lipid mediators may help identify patients with impaired microbial metabolic activity (92, 260, 261). These markers may also help distinguish patients who are more likely to benefit from dietary fiber, prebiotics, butyrate-based approaches, tryptophan-metabolism-directed strategies, probiotics, or postbiotic preparations (191, 260). However, such markers still require validation in well-characterized RA cohorts before they can be used to guide treatment.

Immune phenotyping should also be incorporated into future studies of the gut–joint axis. The Th17/Treg balance, T follicular helper and T follicular regulatory cell phenotypes, monocyte activation, cytokine profiles, autoantibody status, and mucosal IgA responses to candidate pathobionts may all provide information about the immunological status of the patient (6, 262–264). These parameters may be especially useful when analyzed together with barrier and microbiome measurements. At present, however, they remain research tools rather than established clinical biomarkers, and their interpretation must take disease activity and current treatment into account (20, 91, 258, 265).

Difficult-to-treat RA may be one setting in which this type of approach becomes particularly relevant. Some studies suggest that refractory disease may be associated with altered gut microbial profiles and changes in functional pathways related to aromatic compound degradation, mucosal homeostasis, and barrier maintenance (241, 266–268). These observations do not demonstrate that dysbiosis causes treatment resistance; however, they raise the possibility that persistent microbial or metabolic abnormalities may contribute to inflammatory persistence in a subgroup of patients. Such patients may be suitable candidates for exploratory trials in which standard optimization of RA therapy is combined with carefully selected dietary, microbial, metabolic, or exercise-based interventions (241, 266).

The available evidence indicates that the gut–joint axis is a biologically plausible and clinically relevant area of research in RA, although it cannot yet be regarded as an established therapeutic target. At present, the most balanced interpretation is that intestinal barrier dysfunction, altered microbial metabolism, and mucosal immune disturbance may contribute to RA in specific clinical or biological contexts (13, 236, 237). Progress in this field will depend on longitudinal human studies, biomarker-based clinical trials, and the combined assessment of the microbiota, microbial metabolites, epithelial barrier function, and immune responses. Such studies are needed to determine whether interventions directed at the gut may improve disease control, support the maintenance of stable remission, or benefit selected patients with difficult-to-treat RA (14, 236, 241, 243, 266).

### 6.3 Limitations

Despite the increasing interest in the gut–joint axis, a number of limitations need to be considered when interpreting the available evidence. Most studies performed to date have included relatively small cohorts and have been cross-sectional in design. Their results also differ according to geographical background, treatment status, and the analytical methods used. Therefore, in the absence of well-designed longitudinal studies, dysbiosis should still be regarded as an association with RA rather than as evidence of a causal relationship (13, 77, 236).

Lifestyle and treatment-related factors represent an additional source of variation. Diet, proton-pump inhibitors, antibiotics and smoking may affect the gut microbiota or epithelial barrier function, while NSAID exposure may contribute to increased intestinal permeability and intestinal injury (38–40). Treatment with DMARDs may also partially restore altered microbial profiles. As a result, it remains difficult to distinguish microbial changes that may contribute to disease development from those that appear secondary to established inflammation or to its treatment (250, 254, 269–271). Treatment exposure is a major limitation in interpreting RA microbiome studies. The observation that RA-associated oral and gut microbial alterations may be partly normalized after DMARD therapy indicates that microbiome profiles may reflect not only disease biology, but also the effects of treatment (272). Future studies should therefore distinguish disease duration from treatment status, identify patients with newly diagnosed, treatment-naïve RA, and document previous and current medication exposure together with any changes during follow-up. Methodological differences also limit the interpretation of current data. Studies have used different DNA extraction procedures, sequencing platforms, and bioinformatic pipelines, which makes direct comparison between cohorts difficult and reduces the reliability of meta-analysis. In addition, much of the mechanistic evidence still comes from animal models, while human studies remain largely associative. Interventional studies using probiotics, synbiotics, SCFAs, or FMT are also limited in number, generally small, and heterogeneous in design, which means that firm conclusions cannot yet be drawn (209, 242, 273–275).

Similar caution is required when interpreting studies examining the effects of physical exercise on the gut microbiota. Dietary intake is rarely controlled in sufficient detail, and many experimental studies have relied on voluntary exercise models. Therefore, observed changes in microbial composition may reflect differences in nutrition rather than the direct effect of exercise itself. The fact that microbiota responses may also depend on exercise intensity adds a further level of complexity (209, 211, 217, 273–276).

Overall, current research on the gut–joint axis remains at an exploratory stage. Larger, better controlled, and longitudinal studies are needed to clarify whether the observed microbial alterations are involved in the pathogenesis of RA, and to determine whether gut-directed approaches may have therapeutic value in this disease.

## 7 Conclusions and future directions

Recent studies suggest that the intestinal barrier and the gut microbiota are closely related to the development of RA. Experimental models have shown that dysbiosis and impaired epithelial integrity may promote systemic immune activation and contribute to arthritis development. In patients with RA, increased concentrations of biomarkers associated with intestinal permeability have also been reported. Moreover, interventions aimed at restoring barrier function, either by strengthening TJs, modifying the gut microbiota, or reducing intestinal inflammation have been shown to attenuate arthritis in animal models. However, evidence from human studies remains largely associative (258, 277–279).

At present, the clinical translation of these findings is still at an early stage. Ongoing studies assessing probiotic supplementation, FMT, and dietary interventions in RA may help to determine whether gut-directed therapies can provide measurable benefits in patients. Gut-derived biomarkers may help identify individuals at increased risk for RA, although prospective studies are needed to establish how well they predict disease development (17, 280–282). Blood-based measurements provide only indirect information about the intestinal barrier. They should therefore not be interpreted as direct measures of permeability, particularly when they rely on zonulin-related assays with limited specificity (28, 29).

In the future, an integrated therapeutic approach combining conventional immunosuppression with interventions aimed at improving gut barrier function may become a useful direction in RA management. Such strategies could include dietary modification, targeted microbiome-based interventions, and structured physical activity (13, 237, 283, 284).

Baseline microbial composition and function, ZFPs and fecal metabolite profiles may be evaluated as candidate markers of treatment response. Their value for patient selection should be tested against clinical outcomes, since changes in microbial or barrier-related measurements alone do not establish a benefit in RA (263, 285–289).
